# Supplementary material for: Explaining variations in test ordering in primary care: protocol for a realist review
Source: BMJ Open. 2018 Sep 12;8(9):e023117. doi: 10.1136/bmjopen-2018-023117 (PMC6144329; doi:10.1136/bmjopen-2018-023117)
Supplement: Supplementary data [file bmjopen-2018-023117supp001.pdf]

Growth in ‘overdiagnosis’ literature

This chart shows the dramatic increase in published literature on the topic of ‘overdiagnosis’ since the late 2000s as awareness of the problem has grown.

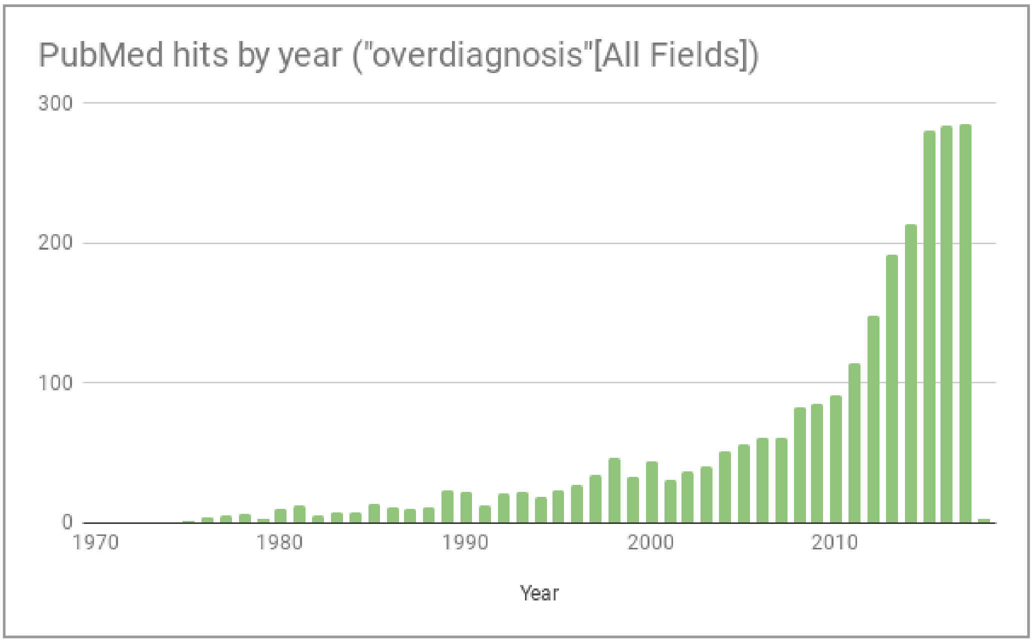

A specific MeSH term, “Medical Overuse”, covering overdiagnosis, overtreatment and related ‘too much medicine’ concepts was introduced in 2016, another sign of increasing interest (previously articles on this topic were indexed as part of the broader “Unnecessary Procedures” category (introduced in 1997).

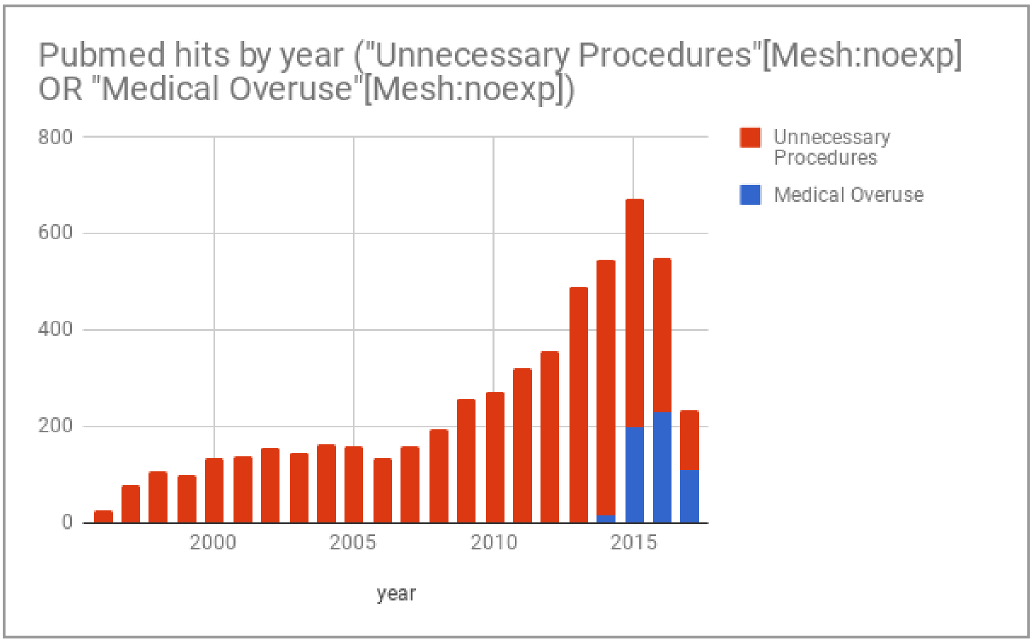

Glossary of realist terminology
(adapted from Realist Synthesis: RAMESES Training Materials)[1]

CMOC	"Context-mechanism-outcome configuration"; a diagrammatic or narrative description offering an explanation of the relationship between some particular context(s), mechanism(s) and outcome(s).<br><br>Multiple CMOCs may exist within a single programme theory.
Context	"Any condition that triggers and/or modifies the behaviour of a mechanism"[2]<br><br>Context refers to the important feature(s) of the circumstances in which a programme exists (or a phenomenon happens) which 'trigger' the mechanisms that generate outcomes. Changes in context over time or in different settings will affect whether and which mechanisms are in operation.
Demi-regularity	A semi-predictable pattern, such as we expect to observe in outcomes that depend on human choice or agency.[2]
Folk theory	An informal theory describing how an intervention is assumed or known to work, or how a phenomenon is assumed or known to come about, according to those practitioners or participants directly involved
Mechanism (generative)	The underlying process by which outcomes are generated. Mechanisms are usually descriptions of the tendencies, reasoning and behaviour of agents involved in a process or participants in a programme and their response to the important context(s) in which they exist.<br><br>Mechanisms are distinguished from "variables or correlates"[3] that are associated with particular outcomes; instead they offer an explanation for why and how observed outcomes happen.
Middle-range theory	A theory that is specific enough to be tested (e.g. against secondary evidence in a realist review, or against primary evidence in a realist evaluation), but abstract or generalisable enough to be transferable, and have explanatory value in other situations.
Programme theory	A theory that describes what an intervention comprises and how it is expected to work, or the process by which the outcomes of interest are thought to come about (expressed as a narrative description or in a diagram).<br><br>A realist programme theory is expressed in terms of the relationship between relevant context(s), mechanism(s) and outcome(s) (or CMOCs).
Substantive theory	An existing established theory from any discipline that can be used to help understand the intervention or phenomenon under examination.

Strategies for early theory-seeking searches

PubMed (19/12/2017)

1	((overdiagnos* OR over-diagnos* OR overtreat* OR over-treat* OR over-prescri* OR overprescri* OR "Unnecessary Procedures"[Mesh])) AND ((("logic model" OR "theory of change" OR "theory of action" OR "outcomes chain" OR "program* theory" OR "program* logic" OR "logical framework*")))	16 hits
2	(overtest* OR over-test* OR “inappropriate test*” OR “unnecessary test*” OR “irrational test*”) AND ("logic model" OR "theory of change" OR "theory of action" OR "outcomes chain" OR "program* theory" OR "program* logic" OR "logical framework*")	5 hits
3	((overdiagnos* OR over-diagnos* OR overtreat* OR over-treat* OR over-prescri* OR overprescri* OR "Unnecessary Procedures"[Mesh])) AND (("theor*" OR "framework*" OR "concept*" OR "model*"))	999 hits
4	(overtest* OR over-test* OR “inappropriate test*” OR “unnecessary test*” OR “irrational test*”) AND (theor* OR framework* OR concept* OR model*)	154 hits

Google Scholar (19/12/2017)

(overdiagnosis OR Overtreatment OR overtesting OR "unnecessary test" OR "inappropriate test" OR "irrational test") AND (theory OR framework OR concept* OR model*)
42,100 hits (first 15 pages screened)

(overdiagnosis OR overtreatment OR overtesting) AND ("logic model" OR "theory of change" OR "theory of action" OR "outcomes chain" OR "program theory" OR "program logic" OR "logical framework")
164 hits

References

1 Wong G, Westhorp G, Pawson R, *et al. Realist Synthesis: RAMESES Training Materials*. 2013. http://www.ramesesproject.org/media/Realist_reviews_training_materials.pdf (accessed 7 Dec 2017).
2 Jagosh J, Macaulay AC, Pluye P, *et al.* Uncovering the Benefits of Participatory Research: Implications of a Realist Review for Health Research and Practice. *Milbank Q* 2012;**90**:311–46. doi:10.1111/j.1468-0009.2012.00665.x
3 Pawson R, Tilley N. *Realistic evaluation*. London: : Sage 1997.
