# Supplementary material for: Explaining variations in test ordering in primary care: protocol for a realist review
Source: BMJ Open. 2018 Sep 12;8(9):e023117. doi: 10.1136/bmjopen-2018-023117 (PMC6144329; doi:10.1136/bmjopen-2018-023117)
Supplement: Author's manuscript [file bmjopen-2018-023117.draft_revisions.pdf]

BMJ Open

BMJ Open is committed to open peer review. As part of this commitment we make the peer review history of every article we publish publicly available.

When an article is published we post the peer reviewers' comments and the authors' responses online. We also post the versions of the paper that were used during peer review. These are the versions that the peer review comments apply to.

The versions of the paper that follow are the versions that were submitted during the peer review process. They are not the versions of record or the final published versions. They should not be cited or distributed as the published version of this manuscript.

BMJ Open is an open access journal and the full, final, typeset and author-corrected version of record of the manuscript is available on our site with no access controls, subscription charges or pay-per-view fees (http://bmjopen.bmj.com).

If you have any questions on BMJ Open's open peer review process please email info.bmjopen@bmj.com

BMJ Open

Explaining variations in test ordering in primary care: protocol for a realist review.

Journal:	*BMJ Open*
Manuscript ID	bmjopen-2018-023117
Article Type:	Protocol
Date Submitted by the Author:	21-Mar-2018
Complete List of Authors:	Duddy, Claire; University of Oxford, Nuffield Department of Primary Care Health Sciences<br>Wong, Geoffrey; University of Oxford, Nuffield Department of Primary Care Health Sciences
Keywords:	PRIMARY CARE, PATHOLOGY, Quality in health care < HEALTH SERVICES ADMINISTRATION & MANAGEMENT

SCHOLARONE™
Manuscripts

For peer review only

Explaining variations in test ordering in primary care: protocol for a realist review.

Duddy, Claire* and Wong, Geoff

***Corresponding author**
Claire Duddy

Nuffield Department of Primary Care Health Sciences
Radcliffe Observatory Quarter
Woodstock Road
Oxford OX2 6GG

claire.duddy@phc.ox.ac.uk
+44 (0)1865 289300

Other authors
Geoff Wong
Nuffield Department of Primary Care Health Sciences, University of Oxford, Oxford, UK

Word count (excluding title page, abstract, references, figures, tables): 7866

For peer review only

ABSTRACT

Introduction

Studies have demonstrated the existence of significant variation in test ordering patterns in both primary and secondary care, for a wide variety of tests and across many health systems. Inconsistent practice could be explained by differing degrees of under- and overuse of tests for diagnosis or monitoring. Underuse of appropriate tests may result in delayed or missed diagnoses; overuse may be an early step that can trigger a cascade of unnecessary intervention, as well as being a source of harm in itself.

Methods and analysis

This realist review will seek to improve our understanding of how and why variation in laboratory test ordering comes about. A realist review is a theory-driven systematic review informed by a realist philosophy of science, seeking to produce useful theory that explains observed outcomes, in terms of relationships between important contexts and generative mechanisms.

An initial explanatory theory will be developed in consultation with a stakeholder group and this 'programme theory' will be tested and refined against available secondary evidence, gathered via an iterative and purposive search process. This data will be analysed and synthesised according to realist principles, to produce a refined 'programme theory', explaining the contexts in which primary care doctors fail to order 'necessary' tests, and/or order 'unnecessary' tests, and the mechanisms underlying these decisions.

Ethics and dissemination

Ethical approval is not required for this review. A complete and transparent report will be produced in line with the RAMESES standards. The theory developed will be used to inform recommendations for the development of interventions designed to minimise 'inappropriate' testing. Our dissemination strategy will be informed by our stakeholders. A variety of outputs will be tailored to ensure relevance to policy makers, primary care and pathology practitioners, and patients.

Keywords: realist review, variation, test ordering, primary care

For peer review only

Strengths and limitations of this study

- First realist review exploring how, why and in what circumstances variations in test ordering in primary care come about;
- Realist approach embraces complexity, seeking to develop understanding of multiple causes of variation and to explore the role of different contexts;
- Involvement of stakeholders in refining programme theory and disseminating outputs will ensure relevance and applicability;
- Availability and richness of available evidence may limit theory building.

BACKGROUND

Variation in test ordering

A large number of studies and reports have demonstrated the existence of significant variation in primary and secondary care test ordering patterns, across many different health systems.[1–15] This variation in practice could be explained by differing degrees of under- and overuse of diagnostic testing in these different settings. Primary studies and reviews that attempt to assess the extent of 'inappropriate' test use usually assess observed test use against chosen guideline standards.[16,17] This approach has limitations, as assessments can only be made wherever guidelines or protocols exist, and will only be as reliable as the guidelines themselves.

This review is concerned with the use of laboratory tests in primary care settings. Our initial focus will be on the NHS in the UK, but we will endeavour to develop recommendations relevant in other settings and countries, where it is likely that the same mechanisms and contexts produce similar outcomes. The use of such tests in UK primary care is extensive, and growing,[10] and is known to vary substantially by region.[8,10] In 2006, the Carter Review reported that 35-45% of requests for laboratory tests in the UK came from primary care.[18] Although an individual laboratory test may be inexpensive, high volumes mean that overall expenditure is high. The same review estimated that pathology services cost the NHS around £2.5 billion per year.[18]

Undertesting and overtesting

Although variations in test ordering practice clearly occur, categorising this practice as under- or overtesting can be more difficult. As noted above, existing studies usually rely on assessing test ordering behaviour against existing guideline or protocol standards. For individual patients, it may only be possible to decide that a particular testing decision was 'inappropriate' later, in light of the results and subsequent decisions (and in many cases, this may be impossible to ascertain even then.[19,20] The picture is further complicated by the possibility that under- and overtesting may occur simultaneously.[21]

It is clear however that both under- and overtesting can have negative consequences for patients. Underutilisation of appropriate tests can result in delayed, missed or incorrect diagnoses and subsequent treatment, and failure to appropriately monitor patients with existing conditions can also result in harm. Uneven access to tests and treatment for different population groups is also a concern.[22–24]

For peer review only

Overtesting is also a problem. Overdiagnosis and consequent overtreatment are increasingly seen as an important source of harm within many healthcare systems. The phenomenon of 'too much medicine' is considered by many to result in direct and indirect harm to individual patients in the form of unnecessary labelling and treatment[25–28] as well as posing a threat to sustainability and equity in healthcare systems, increasing costs[29,30] and diverting resources from the genuinely ill to the 'worried well'.[31]

The increasing interest in this area is reflected in campaigning, including the BMJ's 'Too Much Medicine'[32] (launched in 2002) and 'Choosing Wisely'[33] (launched in the UK in 2016), in a growing number of popular books[34–37] and articles in the mainstream media,[38–41] and in a rapidly growing literature (see Supplementary File). A recent wide-ranging (though not systematic) review[42] drew attention to the large number of 'drivers' of medical overuse that have been identified, but also highlighted the limitations of the existing literature, which is dominated by "analyses or commentaries".[42]

Medical overuse, including overtesting, is often considered under the 'overdiagnosis' banner. Precise definitions are contested,[19,43,44] but terminology like 'overdiagnosis' is frequently used broadly by both researchers and activists to cover a wide range of issues. A broad conceptualisation encompasses concerns ranging from the over-detection of harmless cancers during screening (and their subsequent overtreatment)[45] to widening definitions of disease and pre-disease,[28,46] and many more. The common thread is the identification of medical care that is provided despite "a low probability of benefiting the person diagnosed"[47] and indeed, the possibility that such care may instead be a source of harm.

'Overtesting' may therefore be defined in these terms, as the use of tests where there is a low probability that test results will benefit the patient. This could be the case where there is a lack of evidence to support the use of a test, the use of tests where their results are unlikely to change subsequent management or unnecessary repeat test ordering. Conversely, 'undertesting' may occur in the opposite circumstances.

Overdiagnosis and overtreatment phenomena are usually quantified only at population level.[44,48] However, outcomes of under- and overtesting are the cumulative effect of many individual decisions taken in a variety of circumstances, within the social system of healthcare. A preliminary map of the decisions faced by both patients and doctors in a primary care setting, alongside some important contextual considerations, is provided below in Figure 1.

[Figure 1 here]

The decision to order tests is an important feature of this process and an over-reliance on testing has been identified as an important early step that may result in a cascade of further testing and intervention, including the potential for overdiagnosis and overtreatment.[35,49,50] In addition, overtesting and its consequences can directly increase anxiety and worry for

For peer review only - http://bmjopen.bmj.com/site/about/guidelines.xhtml

patients[51–53] and commentators have highlighted the limited capacity of even ‘gold standard’ tests in providing definitive diagnostic answers.[44]

Existing reviews

Two existing systematic reviews assess ‘inappropriate’ under- and overtesting in secondary[9] and primary[7] care settings: both identified significant variation in practice across a wide range of tests and settings. One health technology assessment considers the extent and consequences of routine pre-operative testing.[54] In addition, several systematic reviews assess the efficacy of various interventions designed to reduce variability and improve ‘appropriateness’ of test ordering in a wide variety of settings.[55–67] One review considers a wide range of variables associated with ‘test-ordering tendencies’.[68]

No realist reviews on this subject have been found. The wide variation in test-ordering behaviour, and in the outcomes of studies aiming to reduce ‘inappropriate’ testing indicates that an enquiry into the role of context could have explanatory value for this phenomenon. Patterns of test-ordering behaviour may vary in response to important contextual factors, such as those highlighted in Figure 1 above. A number of existing studies have highlighted the wide variety of potential drivers of variation in practice, including clinician and patient characteristics[68,69] and health system characteristics.[2,6,14,68,70] A realist review of the literature will allow consideration of multiple causal mechanisms, sensitivity to context and opening the ‘black box’[71] of decision-making in relation to ordering tests.

Realist review

A realist review (otherwise known as ‘realist synthesis’) is a type of theory-driven systematic literature review. Originally proposed as a means to explore the inner workings of similar ‘families’ of complex social interventions,[72] its utility in helping to ‘diagnose’ and understand the underlying nature of complex problems has also been established.[73,74] For a glossary of realist terminology, see Supplementary File.

Here, the overall problem of medical overuse and the specific issues of over- and undertesting are characterised as ‘complex’: the literature suggests multiple potential causes operating at different levels, as well as potential emergent effects, whereby (for example) more testing begets even more testing,[25,75] and variable outcomes exist (underdiagnosis and overdiagnosis coexist in the same healthcare system, for example).[76] Decisions to order tests in primary care are made within the context of the interaction between provider and patient; as such there are multiple opportunities for the reasoning and behaviour of both parties to influence the outcome.[77]

The realist approach is underpinned by a realist understanding of the world: observed outcomes (such as medical overuse) are understood to be the product of underlying, hidden ‘mechanisms’ which may operate differently (or not at all) in different contexts.

‘Mechanisms’ are understood as the causal forces of patterns of observed outcomes (or ‘demi-regularities’) that have their roots in individual tendencies and reasoning.[72] Causation is

For peer review only

‘generative’, that is, outcomes in social systems are not the direct result of interventions or simple responses to stimuli, but rather reflect the invisible reasoning and behaviour of actors within those systems.[78] Such reasoning may change (or not) in different contexts, where different resources are available to different actors with different capacities to respond to their circumstances. The realist approach can allow us to go beyond an assessment of those variables associated with a particular outcome, to shed light on the real generative mechanisms that are the underlying causes of observed test-use, and to highlight the context(s) or conditions in which these mechanisms operate.[79]

Realist inquiry begins (and ends) with a ‘programme theory’, describing a hypothesis about how an intervention works or how a phenomenon comes about. Realist programme theories are models that describe relationships between important contexts, mechanisms and outcomes, usually presented and described as sequences of ‘context-mechanism-outcome configurations’ (‘CMOCs’). Such configurations aim to explain in which context(s), which mechanism(s) are ‘triggered’ to produce which outcomes(s). As such, the realist approach is especially useful where outcomes appear to vary with circumstances, seeking to provide explanatory evidence for such variation, and offers a means of adjudicating between competing theories and/or refining and improving an initial theory to accommodate multiple explanatory mechanisms.[79]

A realist programme theory should be in the ‘middle range’, i.e. it should be specific enough to permit empirical testing (in this case, against secondary evidence located during the review process), but abstract enough to provide useful, explanatory transferability to other situations where the same mechanisms may be operating.[80]

REVIEW OBJECTIVES AND DESIGN

Review Objectives

1. Develop a realist programme theory offering explanation(s) for the variation in test ordering in primary care, underpinned by secondary evidence.
2. Make recommendations based upon this explanation, to inform the design of existing and new interventions that could help to reduce this problem.

Review Questions

1. How are ‘undertesting’ and ‘overtesting’ conceptualised in the literature?
2. In what contexts do primary care doctors order ‘unnecessary’ tests?
3. In what contexts do primary care doctors fail to order ‘necessary’ tests?
4. What mechanisms are at work in these different contexts that underlie test-ordering behaviour and generate these outcomes?

The review will be conducted according to Pawson’s five stages[81,82] which outline the processes by which an initial programme theory will be developed, evidence gathered and refinements to theory made. The RAMESES quality[83] and reporting[84] standards will be followed. Figure 2 summarises the overall project design, and more details on each step are provided below.

For peer review only

A “guiding principle” of the realist approach is the maintenance of transparency of methods and decision-making throughout the review.[84] Such transparency ensures that the iterative nature of the research is made clear and that decisions taken in consultation with stakeholders and within the project team are fully explained and justified. Such decisions determine the direction and focus of the project, as well as guiding the extent and direction of literature searching, and the analysis and synthesis themselves.

[Figure 2 here]

Stakeholder involvement

Following an established approach,[74] a diverse stakeholder group will be recruited at the beginning of the project. This group will include, for example, primary care clinicians, pathologists, managers and policymakers. The involvement of stakeholders at multiple stages is made clear in Figure 2. This group will provide the content expertise essential for initial programme theory development. Stakeholders may also suggest useful sources of evidence, and members of the group will be asked to provide feedback on iterations of refined programme theory as these are developed.

Step 1: Develop initial programme theory

The first stage of a realist review is the development of an ‘initial programme theory’ which makes a first attempt to explain the phenomenon under examination. The development of this theory will be informed by two main processes: an informal scoping search of the literature, and input from the stakeholder group.

Iterative, informal searching will be used to locate existing theories that are used to explain how and why overtesting and undertesting occur. This initial search stage will rely on a combination of more structured searching[85,86] and more emergent techniques such as reference and citation tracking (‘snowballing’) and personal contacts.[87] An inclusive approach will be used to screen documents found at this stage, with no limitations placed on type of study or document. Documents will be selected wherever there is an attempt to theorise about the causes of variation in test ordering, especially in relation to the circumstances in which such variation is most prevalent, and the reasoning of actors involved (even where such ideas are not identified formally as ’theory’).

This process may uncover informal ‘folk theories’[88] attempting to explain the causes of variations in practice, and theories that underpin actual and proposed interventions designed to reduce the problem,[72] as well as potentially useful ‘substantive’ theory,[89] i.e. established theory from any discipline which can help to explain the phenomenon. The stakeholder group will also be consulted to ensure that their content expertise is used to supplement the results of this early searching. Candidate initial programme theories will be presented, and stakeholders asked to provide feedback and commentary on their plausibility and ‘fit’ with their experience. Through this process, initial theory(ies) are likely to be refined and prioritised for the next stage of the review.

Work on this stage has begun and is ongoing. Initial search strategies focused on identifying relevant substantive theories are available in the Supplementary File. Figure 3 below illustrates the basis of an early set of initial programme theories, considering the 'decision to order test(s)' step from Figure 1 above.

[Figure 3 here]

Initial exploration of the literature has uncovered a range of potentially useful substantive theory that could help to explain the mechanisms underlying the decision-making involved in test-ordering behaviour, including economic theory explaining over-supply and over-consumption in "experts markets",[90] theories of decision-making that assume bounded rationality[91], including regret theory[92] and threshold models[93] and several others.[94–96] These theories can be explored in relation to their ability to provide a useful lens through which to view this decision-making process in a realist fashion and explain observed outcomes. For example, 'regret theory' suggests the possibility of an underlying mechanism related to the estimation and minimisation of 'expected regret' in deciding to order a test or otherwise.

Another potentially valuable source in the development of initial programme theory are those theories underlying interventions designed to reduce overtesting. Instead of assuming a complex decision-making process is happening, many such interventions seem based on the theory that test-ordering is at least to some extent a habitual, normalised behaviour[97] and so seek to disrupt these habits. For example, interventions designed to increase barriers to test-ordering[98,99] may create space for doctors to consider whether a test is really necessary. Similarly, interventions designed to promote reflective practice[100–102] provide opportunities for doctors to reflect on their past test-ordering behaviour and outcomes and potentially change their behaviour in the future. Interventions based around computer-aided decision-support systems[103,104] may seek to replace old habits with new, evidence-based ones.

These initial theories can be conceptualised in a 'realist' fashion (i.e. in the form of a CMOC), as illustrated in the hypothesised example in Figure 4 below.

[Figure 4 here]

The candidate theories uncovered during searching will be considered by the project team alongside Figure 3 to refine these initial CMOCs. These will be discussed with the stakeholder group and refined as necessary in light of these discussions and further reading. It is likely that a small number of candidate theories will be prioritised as a focus for the review, based on their greater importance and/or resonance with stakeholders.

Step 2: Searching for evidence

Secondary evidence gathered in cycles over the course of a realist review is iteratively interpreted and used to "confirm, refute or refine" each aspect of a programme theory.[105] This evidence is sought from a wide range of sources and disciplines: there is no 'hierarchy of

For peer review only

evidence' in a realist approach and so evidence may include quantitative and qualitative data, peer-reviewed articles, opinion and commentary, and grey literature like policy documents.[89]

The main systematic literature search(es) will be conducted with the aim of identifying relevant documents potentially containing data that can be used to develop or refine, refute or confirm, the initial programme theory(ies) chosen for testing.

A search strategy(ies) will be designed, piloted and executed by an information specialist (CD). A wide range of bibliographic databases covering multiple disciplines will be considered for searching, including MEDLINE, Embase, CINAHL, PsycINFO, PsycEXTRA, the Web of Science Core Collection, Scopus, ASSIA, IBSS, EconLit and Google Scholar. Sources of grey literature will be searched, including via web search engines. Free text and subject heading search terms will be chosen as appropriate, and the search strategy will be refined iteratively to achieve a balance of sensitivity and specificity. As for the informal search stage, 'snowballing' and other supplementary search techniques will be used to identify additional documents.[87]

Search results will be screened initially by title and abstract, with full text considered as a second step. A broad set of inclusion and exclusion criteria will be used to screen the results of the main search. These criteria will be finalised when the initial programme theory is confirmed, but are likely to include some or all of the following:

Inclusion criteria

- All types of document;
- Any study design;
- Studies or documents that identify variation in test use, actual or potential under- or overuse of tests, or are focused on areas of primary health care where under- or overtesting is a recognised problem;
- Studies or documents focused on primary care settings;
- If a particular type of test or specific test is chosen as a focus in consultation with the stakeholder group, searching may initially be limited to consider this area.

Exclusion criteria

- Studies or documents focused on secondary care settings (though searches may be broadened later to consider additional settings if there is a dearth of literature focused on primary care, or where the stakeholder group or initial searches suggest common mechanisms may be in operation);
- Studies focused on imaging, genetic testing, foetal monitoring, near-patient testing, self-testing, or home-based testing by patients (though searches may be broadened later, as above);
- Studies or documents focused on low and middle income settings, where limited resources are likely to create very different contextual factors that are outwith the scope of this review.

For peer review only

Screening of titles and abstracts will be undertaken first by one reviewer; a 10% random sample of search results will be screened by the second reviewer (GW) to check for consistency. Disagreements will be recorded and resolved via discussion in the project team.

As Figure 2 illustrates, additional searching may be undertaken as required at later stages of the review, wherever the main search did not generate sufficient data to test programme theory (e.g. if data on particular contexts or mechanisms was sparse), or in response to potential programme theory refinements. All such additional searches will be developed with an information specialist and screened as described above.

All searching and screening processes will be reported in full, including PRISMA-style flow diagrams,[106] to ensure transparency of evidence sources.

Step 3: Selection and appraisal

Following screening, documents will be selected on the basis of an assessment of their relevance (i.e. whether some part(s) of the document can contribute to the refinement of programme theory) and rigour (i.e. the trustworthiness of that data).[82] One reviewer (CD) will read all of the documents that met the inclusion criteria during screening and assess their ability to speak to some aspect of the programme theory under consideration (i.e. relevance). Relevant data from these documents will then be assessed for rigour.

The assessment of rigour in a realist review is not conducted at article- or document-level as in a 'traditional' review, since doing so may exclude documents containing relevant data[89] and even where a study as a whole is methodologically weak in terms of its own objectives, it may still contain 'nuggets' of useful data.[107] Instead, each piece of relevant contributing data will be judged according to its purpose in testing programme theory[82] and the methodology by which the particular piece of data was produced. This may involve the use of formal critical appraisal checklists suitable for different study types, but only as one part of determining trustworthiness. Different types of data will be subject to different judgements of methodological coherence and plausibility,[89] and the details of each assessment will be recorded in full to ensure that this process is transparent.

As with screening, a 10% random subsample of documents will be assessed by a second reviewer (GW) using the same criteria, with disagreements recorded and resolved via discussion in the project team. It anticipation of uncertainty in the case of some documents, the project team may also be called upon to make assessments as a group.[74,108]

Step 4: Extracting and organising data

One reviewer will extract the main characteristics of each included document into an Excel spreadsheet. The full text of all of the documents will then be uploaded into the NVivo QRS International qualitative data analysis tool. One reviewer will then organise and classify this data, by annotating (coding) relevant data from each document according to its contribution to the developing programme theory.[82]

For peer review only

The initial phase of organising and coding data will be informed by any contexts, mechanisms and outcomes (or concepts not yet clarified as C, M or O) identified in the development of the initial programme theory. As data extraction progresses, organisation and coding is likely to evolve and include new concepts that reflect refinements to programme theory. As such, each document may be subject to several readings. As noted above, an individual document may include sections that contribute to several elements of programme theory. The use of data to refine programme theory will be recorded, to enable transparent reporting and the inclusion of relevant document extracts within the synthesis.[82] A 10% random subsample of documents that have been through the data extraction and organising process will be reviewed by a second reviewer (GW) to check for consistency, with disagreements recorded and resolved via discussion in the project team.

Step 5: Analysis and synthesis

In a realist review, analysis and synthesis of the selected data proceed in parallel, and will begin at the same point as document selection and appraisal for relevance and rigour, and data extraction and organisation.[79] All three stages may thus proceed simultaneously (see Figure 2), as data are chosen, assessed, annotated and organised according to its potential role in refining the developing programme theory.

This process will be iterative[79]: the programme theory will be refined in stages as more and more data are considered. The stakeholder group will be consulted at various points to obtain feedback on the focus and development of the programme theory and the project timeline will permit pauses in analysis and synthesis for this purpose, and to allow further searching to be undertaken where gaps in the available secondary evidence are found.

Pawson suggests that realist analysis and synthesis should be a process of “juxtaposing, adjudicating, reconciling, consolidating and situating the evidence” in an effort to refine programme theory.[82] As such, data relating to different aspects of the programme theory will be collected together and considered alongside each other, such that an assessment of the strength of evidence supporting the arguments that underpin each aspect of that theory can be made. A process of retroductive reasoning will then be applied, so that refinements to programme theory are made on the basis of what can plausibly be inferred by all the data available. Retroductive reasoning will be used to build explanatory realist theory(ies). This involves an interpretive process of considering which underlying causal mechanisms must be at work to deliver the observed patterns out of outcomes. The approach involves moving back and forth between concrete observations and theory-building, and hence between inductive and deductive reasoning.[109]

LIMITATIONS AND RISKS

An important potential limitation of this study will be the availability and contextual richness of the secondary evidence that is available.[79] Although initial scoping searches suggest that a significant amount of material on the subject of laboratory test-ordering does exist, it is possible that this material will not describe contextual factors in great detail or include enough relevant information on which to build theory. We will attempt to address this problem by ensuring that

comprehensive and wide-ranging searching is undertaken by an information professional, that supporting and related information for all included studies is located wherever it exists,[86] and by contacting authors to ask for further detail as required.

In addition, there are important limitations that are inherent to the nature of the realist review. In particular, there is a limit to how much ground a single review can cover and so this review will necessarily prioritise certain elements of the process within which test ordering takes place[79] and will inevitably have to set aside some potentially important factors for future research. The final output of the review will be a (refined) theory that attempts to illuminate important contextual factors and underlying mechanisms; it is important to acknowledge that such theory can only ever represent partial knowledge that will be open to further refinement or refutation in the future.

OUTPUTS AND DISSEMINATION

A variety of project outputs are planned, to meet the needs of different groups, including national and local policy makers, leaders, employers and practitioners in primary care and pathology settings, and patients. To some extent, outputs will be guided by the review's conclusions and resulting recommendations that may have relevance in different contexts and at different levels.

The RAMESES reporting standards will be used to produce a complete and transparent report of this review – both for the funder and as a standalone publication.[84] The standalone publication will be for academic audiences and will be submitted as an article to a peer-reviewed journal. Other academic outputs will be prepared for presentation at relevant conferences (e.g. 'Preventing Overdiagnosis,[110] International Realist Conference.)[111]

The final refined programme theory and resulting recommendations will be presented to the stakeholder group (to include policymakers, practitioners and patients) and their opinions will be sought to direct the dissemination strategy for these groups, with the aim of ensuring that important recommendations reach the appropriate decision-makers. We will endeavour in particular to reach policy makers and researchers engaged in the development and evaluation of interventions designed to reduce variation in test ordering, in order that future work in this area can be informed by the new knowledge generated in this review. We envision the production of user-friendly and accessible summaries of the findings and our recommendations and the use of existing networks and social media to promote these outputs to help ensure maximum visibility.

Author contributions

CD conceived the study with input from GW. CD wrote the first draft of this manuscript and GW provided criticism and refinement. CD and GW approved the final version.

Funding statement

Claire Duddy (CD) is funded by a National Institute of Health Research (NIHR) Research Methods Programme Systematic Review Fellowship (NIHR-RM-SR-2017-08-018). Geoff Wong

For peer review only

(GW) and Trish Greenhalgh (TG) are supporting CD in this project. TG is partly funded by the National Institute for Health Research Biomedical Research Centre, Oxford, grant BRC-1215-20008 to the Oxford University Hospitals NHS Foundation Trust and the University of Oxford. GW is partly funded by the NIHR School of Primary Care Evidence Synthesis Working Group.

The views expressed in this publication are those of the author(s) and not necessarily those of the NHS, the National Institute for Health Research or the Department of Health.

Competing interests

CD and GW are both members of the Royal College of General Practitioners (United Kingdom) Overdiagnosis and Overtreatment Group.

GW is an NHS General Practitioner and a member of the NIHR Health Technology Assessment Programme Primary Care Panel.

REFERENCES

1 Larsson A, Palmer M, Hultén G, *et al.* Large Differences in Laboratory Utilisation between Hospitals in Sweden. *Clin Chem Lab Med* 2000;**38**:383–9.https://www.degruyter.com/view/j/cclm.2000.38.issue-5/cclm.2000.056/cclm.2000.056.xml (accessed 19 Dec 2017).

2 Verstappen W, Ter Riet G, Dubois WI, *et al.* Variation in test ordering behaviour of GPs: professional or context-related factors? *Fam Pract* 2004;**21**:387–95. doi:10.1093/fampra/cmh408

3 Chami N, Simons JE, Sweetman A, *et al.* Rates of inappropriate laboratory test utilization in Ontario. *Clin Biochem* 2017;**50**:822–7. doi:10.1016/J.CLINBIOCHEM.2017.05.004

4 Lin DC, Straseski JA, Schmidt RL. Multicenter Benchmark Study Reveals Significant Variation in Thyroid Testing in the United States. *Thyroid* 2017;**27**:1232–45. doi:10.1089/thy.2017.0190

5 *The 2nd Atlas of Variation in NHS Diagnostic Services in England Reducing unwarranted variation to improve health outcomes and value*. 2017. www.gov.uk/phe

6 Sá L, Teixeira ASC, Tavares F, *et al.* Diagnostic and laboratory test ordering in Northern Portuguese Primary Health Care: a cross-sectional study. *BMJ Open* 2017;**7**:e018509. doi:10.1136/bmjopen-2017-018509

7 O'Sullivan JW, Albasri A, Nicholson BD, *et al.* Overtesting and undertesting in primary care: a systematic review and meta-analysis. *BMJ Open* 2018;**8**:e018557. doi:10.1136/bmjopen-2017-018557

8 *The NHS Atlas of Variation in Diagnostic Services*. 2013. https://fingertips.phe.org.uk/profile/atlas-of-variation

9 Zhi M, Ding EL, Theisen-Toupal J, *et al.* The Landscape of Inappropriate Laboratory Testing: A 15-Year Meta-Analysis. *PLoS One* 2013;**8**:e78962. doi:10.1371/journal.pone.0078962

10 Busby J, Schroeder K, Woltersdorf W, *et al.* Temporal growth and geographic variation in the use of laboratory tests by NHS general practices: using routine data to identify research priorities. *Br J Gen Pr* 2013;**63**:e256-66. doi:10.3399/bjgp13X665224

11 Davis P, Gribben B, Lay-Yee R, *et al.* How much variation in clinical activity is there between general practitioners? A multi-level analysis of decision-making in primary care. *J Health Serv Res Policy* 2002;**7**:202–8. doi:10.1258/135581902320432723

For peer review only

12 Taroni F, Siass R, Traversa G, *et al.* The nature, content and interpractice variation of general practice: A regional study in Italy. *Eur J Epidemiol* 1990;**6**:313–8. doi:10.1007/BF00150439
13 Hartley RM, Charlton JR, Harris CM, *et al.* Patterns of physicians' use of medical resources in ambulatory settings. *Am J Public Health* 1987;**77**:565–7. doi:10.2105/AJPH.77.5.565
14 Verstappen W, Ter Riet G, Van Der Weijden T, *et al.* Variation in requests for imaging investigations by general practitioners: A multilevel analysis. *J Health Serv Res Policy* 2005;**10**:25–30. doi:10.1177/135581960501000107
15 Powell EC, Hampers LC. Physician Variation in Test Ordering in the Management of Gastroenteritis in Children. *Arch Pediatr Adolesc Med* 2003;**157**:978. doi:10.1001/archpedi.157.10.978
16 van Walraven C, Naylor CD. Do We Know What Inappropriate Laboratory Utilization Is? A Systematic Review of Laboratory Clinical Audits. *JAMA* 1998;**12**:550–8.https://jamanetwork.com/journals/jama/fullarticle/vol/280/pg/550 (accessed 20 Dec 2017).
17 Hauser RG, Shirts BH. Do We Now Know What Inappropriate Laboratory Utilization Is? An Expanded Systematic Review of Laboratory Clinical Audits. *Am J Clin Pathol* 2014;**141**:774–83. doi:10.1309/AJCPX1HIEM4KLGNU
18 Lord Carter of Coles. Report of the review of NHS pathology services in England. 2006. http://webarchive.nationalarchives.gov.uk/20130123210254/http://www.dh.gov.uk/en/PublicationsandStatistics/Publications/PublicationsPolicyAndGuidance/DH_4137606 (accessed 15 Feb 2018).
19 Hofmann B. Diagnosing overdiagnosis: conceptual challenges and suggested solutions. *Eur J Epidemiol* 2014;**29**:599–604. doi:10.1007/s10654-014-9920-5
20 Raffle AE, Gray JAM. *Screening: evidence and practice*. Oxford University Press 2007.
21 Newman-Toker DE. A unified conceptual model for diagnostic errors: underdiagnosis, overdiagnosis, and misdiagnosis. *Diagnosis* 2014;**1**:43–8. doi:10.1515/dx-2013-0027
22 Carrieri V, Wuebker A. Assessing inequalities in preventative care use in Europe: A special case of health-care inequalities? 2012. https://www.york.ac.uk/media/economics/documents/herc/wp/12_25.pdf (accessed 9 Mar 2018).
23 Catalogue of Bias Collaboration, Banerjee A, O'Sullivan J, *et al.* Diagnostic access bias. Cat. Biases. 2017.https://catalogofbias.org/biases/diagnostic-access-bias/ (accessed 9 Mar 2018).
24 Neal RD, Allgar VL. Sociodemographic factors and delays in the diagnosis of six cancers: analysis of data from the 'National Survey of NHS Patients: Cancer'. *Br J Cancer* 2005;**92**:1971–5. doi:10.1038/sj.bjc.6602623
25 Moynihan R, Doust J, Henry D. Preventing overdiagnosis: How to stop harming the healthy. *BMJ* 2012;**344**:e3502. doi:10.1136/bmj.e3502
26 Woolf SH, Harris R. The harms of screening: New attention to an old concern. *JAMA* 2012;**307**:565–6.https://doi.org/10.1001/jama.2012.100 (accessed 6 Dec 2017).
27 Copp T, Jansen J, Doust J, *et al.* Are expanding disease definitions unnecessarily labelling women with polycystic ovary syndrome? *BMJ* 2017;**358**:j3694. doi:10.1136/bmj.j3694
28 Moynihan R, Glassock R, Doust J. Chronic kidney disease controversy: How expanding definitions are unnecessarily labelling many people as diseased. *BMJ* 2013;**347**:f4298. doi:10.1136/bmj.f4298
29 Berwick DM, Hackbarth AD. Eliminating waste in US health care. *JAMA* 2012;**307**:1513–6. doi:10.1001/jama.2012.362
30 Newman-Toker DE, McDonald KM, Meltzer DO. How much diagnostic safety can we

For peer review only

afford, and how should we decide? A health economics perspective. *BMJ Qual Saf* 2013;**22**:ii11-ii20. doi:10.1136/bmjqs-2012-001616
31 Glasziou P, Moynihan R, Richards T. Too much medicine ; too little care. *BMJ* 2013;**347**:1–2. doi:10.1136/bmj.f4247
32 BMJ. Too Much Medicine. http://www.bmj.com/too-much-medicine
33 Choosing Wisely UK. http://www.choosingwisely.co.uk
34 Moynihan R, Cassels A. *Selling Sickness: How Drug Companies Are Turning Us All Into Patients*. Crows Nest, NSW: : Allen & Unwin 2005.
35 Welch H, Schwartz L, Woloshin S. *Overdiagnosed: Making People Sick in the Pursuit of Health*. Boston: : Beacon Press 2011.
36 McCartney M. *The Patient Paradox: Why Sexed-Up Medicine is Bad For Your Health*. Great Britain: : Pinter & Martin 2013.
37 Welch H. *Less Medicine, More Health: 7 Assumptions that Drive Too Much Medical Care*. Boston: : Beacon Press 2015.
38 Edwards M. Doctors 'over-diagnose'. Telegraph. 2010.http://www.telegraph.co.uk/news/health/8008608/Doctors-over-diagnose.html
39 Welch H. If you feel O.K., Maybe You Are O.K. New York Times. 2012.http://www.nytimes.com/2012/02/28/opinion/over-diagnosis-as-a-flaw-in-health-care.html
40 Gawande A. Overkill. New Yorker. 2015.http://www.newyorker.com/magazine/2015/05/11/overkill-atul-gawande
41 Srivastava R. Do I really need 'the test'? Too many tests could do patients more harm than good. Guardian. 2016.http://www.theguardian.com/commentisfree/2016/sep/07/do-i-really-need-the-test-too-many-tests-could-do-patients-more-harm-than-good
42 Pathirana T, Clark J, Moynihan R. Mapping the drivers of overdiagnosis to potential solutions Searching the literature. *BMJ* 2017;**358**. doi:10.1136/bmj.j3879
43 Carter SM, Rogers W, Heath I, *et al.* The challenge of overdiagnosis begins with its definition. *BMJ* 2015;**350**:h869. doi:10.1136/bmj.h869
44 Rogers WA, Mintzker Y. Getting clearer on overdiagnosis. *J Eval Clin Pract* 2016;**22**:580–7. doi:10.1111/jep.12556
45 Welch HG, Black WC. Overdiagnosis in cancer. *J Natl Cancer Inst* 2010;**102**:605–13. doi:10.1093/jnci/djq099
46 Moynihan R, Heath I, Henry D. Selling sickness: the pharmaceutical industry and disease mongering. *BMJ* 2002;**324**:886–91. doi:10.1136/bmj.324.7342.886
47 McCaffery KJ, Jansen J, Scherer LD, *et al.* Walking the tightrope: Communicating overdiagnosis in modern healthcare. *BMJ* 2016;**352**:i348. doi:10.1136/bmj.i348
48 Hofmann B. Defining and evaluating overdiagnosis. *J Med Ethics* 2016;**42**:715–6. doi:10.1136/medethics-2016-103716
49 Deyo RA. Cascade Effects of Medical Technology. *Annu Rev Public Health* 2002;**23**:23–44. doi:10.1146/annurev.publhealth.23.092101.134534
50 Carpenter CR, Raja AS, Brown MD. Overtesting and the downstream consequences of overtreatment: Implications of 'preventing overdiagnosis' for emergency medicine. *Acad Emerg Med* 2015;**22**:1484–92. doi:10.1111/acem.12820
51 Hofmann B, Welch HG. New diagnostic tests: more harm than good. *BMJ* 2017;**358**:j3314. doi:10.1136/bmj.j3314
52 Fisher ES, Welch HG. Avoiding the Unintended Consequences of Growth in Medical Care. *JAMA* 1999;**281**:446–53. doi:10.1001/jama.281.5.446
53 Harris RP, Sheridan SL, Lewis CL, *et al.* The harms of screening : A proposed taxonomy and application to lung cancer screening. *JAMA Intern Med* 2014;**174**:281–6. doi:10.1001/jamainternmed.2013.12745
54 Czoski-Murray C, Lloyd Jones M, McCabe C, *et al.* What is the value of routinely testing

For peer review only

full blood count, electrolytes and urea, and pulmonary function tests before elective surgery in patients with no apparent clinical indication and in subgroups of patients with common comorbidities: a systematic review of the clinical and cost-effective literature. *Health Technol Assess (Rockv)* 2012;**16**. doi:10.3310/hta16500

55 Beilby J, Silagy C. Trials of providing costing information to general practitioners: a systematic review. *Med J Aust* 1997;**167**:89–92.

56 Georgiou A, Williamson M, Westbrook JI, *et al.* The impact of computerised physician order entry systems on pathology services: A systematic review. *Int J Med Inform* 2007;**76**:514–29. doi:10.1016/J.IJMEDINF.2006.02.004

57 Main C, Moxham T, Wyatt JC, *et al.* Computerised decision support systems in order communication for diagnostic, screening or monitoring test ordering: Systematic reviews of the effects and cost-effectiveness of systems. *Health Technol Assess (Rockv)* 2010;**14**:1–227. doi:10.3310/hta14480

58 Georgiou A, Prgomet M, Markewycz A, *et al.* The impact of computerized provider order entry systems on medical-imaging services: a systematic review. *J Am Med Informatics Assoc* 2011;**18**:335–40. doi:10.1136/amiajnl-2010-000043

59 Roshanov PS, You JJ, Dhaliwal J, *et al.* Can computerized clinical decision support systems improve practitioners' diagnostic test ordering behavior? A decision-maker-researcher partnership systematic review for the CCDSS Systematic Review Team. *Implement Sci* 2011;**6**. doi:10.1186/1748-5908-6-88

60 Cadogan SL, Browne JP, Bradley CP, *et al.* The effectiveness of interventions to improve laboratory requesting patterns among primary care physicians: a systematic review. *Implement Sci* 2015;**10**. doi:10.1186/s13012-015-0356-4

61 Goldzweig CL, Orshansky G, Paige NM, *et al.* Electronic Health Record–Based Interventions for Improving Appropriate Diagnostic Imaging. *Ann Intern Med* 2015;**162**:557–65. doi:10.7326/M14-2600

62 Kobewka DM, Ronksley PE, Mckay JA, *et al.* Influence of educational, audit and feedback, system based, and incentive and penalty interventions to reduce laboratory test utilization: a systematic review. *Clin Chem Lab Med* 2015;**53**:157–83. doi:10.1515/cclm-2014-0778

63 Thomas RE, Vaska M, Naugler C, *et al.* Interventions to Educate Family Physicians to Change Test Ordering. *Acad Pathol* 2016;**3**:237428951663347. doi:10.1177/2374289516633476

64 Delvaux N, Thienen K Van, Heselmans A, *et al.* The Effects of Computerized Clinical Decision Support Systems on Laboratory Test Ordering A Systematic Review. *Arch Pathol Lab Med* 2017;**141**:585–95.http://www.archivesofpathology.org/doi/pdf/10.5858/arpa.2016-0115-RA (accessed 20 Dec 2017).

65 Ho J, Chau J, Cheung N. Effectiveness of emergency nurses' use of the Ottowa Ankle Rules to initiate radiographic tests on improving healthcare outcomes for patients with ankle injuries: a systematic review. *Int J Nurs Stud* 2016;**63**:37–47. doi:10.1016/j.ihnurstu.2016.08.016

66 Thomas RE, Vaska M, Naugler C, *et al.* Interventions at the laboratory level to reduce laboratory test ordering by family physicians: Systematic review. *Clin Biochem* 2015;**48**:1358–65. doi:10.1016/J.CLINBIOCHEM.2015.09.014

67 Ivers N, Jamtvedt G, Flottorp S, *et al.* Audit and feedback: effects on professional practice and healthcare outcomes. *Cochrane Database Syst Rev* 2012;:CD000259. doi:10.1002/14651858.CD000259.pub3

68 Sood R, Sood A, Ghosh AK. Non-evidence-based variables affecting physicians' test-ordering tendencies: a systematic review. *Neth J Med* 2007;**65**:167–77.http://www.njmonline.nl/getpdf.php?id=529 (accessed 20 Dec 2017).

For peer review only

69 Eisenberg JM. Physician Utilization: The State of Research about Physicians' Practice Patterns. *Med Care* 1985;**23**:461–83. doi:10.2307/3764984

70 Leurquin P, Van Casteren V, De Maeseneer J. Use of blood tests in general practice: a collaborative study in eight European countries. Eurosentinel Study Group. *Br J Gen Pract* 1995;**45**:21–5.http://www.ncbi.nlm.nih.gov/pubmed/7779470 (accessed 4 Jan 2018).

71 Wong G, Greenhalgh T, Pawson R. What is a realist review and what can it do for me? An introduction to realist synthesis. 2009.http://pram.mcgill.ca/Wong_G_JUNE09_what_is_a_realist_review_presentation.pdf

72 Pawson R. Realist Methodology: The Building Blocks of Evidence. In: *Evidence-based policy: a realist perspective*. London: : SAGE Publications Ltd 2006. 17–37. doi:10.4135/9781849209120

73 Wong G. 'Wrong diagnosis, wrong treatment': why researchers and clinicians should question their assumptions. NIHR Blog. 2017.https://www.nihr.ac.uk/blogs/wrong-diagnosis-wrong-treatment-why-researchers-and-clinicians-should-question-their-assumptions/7374

74 Papoutsi C, Mattick K, Pearson M, *et al.* Social and professional influences on antimicrobial prescribing for doctors-in-training: a realist review. *J Antimicrob Chemother* 2017;**72**:2418–30. doi:10.1093/jac/dkx194

75 Hofmann BM. Too much technology. *BMJ* 2015;**350**:h705. doi:10.1136/BMJ.H705

76 Petticrew M, Rehfuess E, Noyes J, *et al.* Synthesizing evidence on complex interventions: how meta-analytical, qualitative, and mixed-method approaches can contribute. *J Clin Epidemiol* 2013;**66**:1230–43. doi:10.1016/j.jclinepi.2013.06.005

77 Pawson R. A Complexity Checklist. In: *The Science of Evaluation: A Realist Manifesto*. London: : SAGE Publications Ltd 2013. 33–46. doi:10.4135/9781473913820

78 Pawson R. Invisible Mechanisms I; The Long Road to Behavioural Change. In: *The Science of Evaluation: A Realist Manifesto*. London: : SAGE Publications Ltd 2013. 115–37. doi:10.4135/9781473913820

79 Pawson R, Greenhalgh T, Harvey G, *et al.* Realist review - a new method of systematic review designed for complex policy interventions. *J Health Serv Res Policy* 2005;**10**:21–34. doi:10.1258/1355819054308530

80 Pawson R. Informed Guesswork: The Realist Response to Complexity. In: *The Science of Evaluation: A Realist Manifesto*. London: : SAGE Publications Ltd 2013. 81–112. doi:10.4135/9781473913820

81 Pawson R, Greenhalgh T, Harvey G, *et al.* Realist synthesis: an introduction. 2004. https://www.researchgate.net/profile/Gill_Harvey/publication/228855827_Realist_Synthesis_An_Introduction/links/0fcfd507f0b7cbb2ce000000/Realist-Synthesis-An-Introduction.pdf (accessed 7 Dec 2017).

82 Pawson R. Realist Synthesis: New Protocols for Systematic Review. In: *Evidence-based policy: a realist perspective*. London: : SAGE Publications Ltd 2006. 73–104. doi:10.4135/9781849209120

83 RAMESES Project Team. *RAMESES Quality Standards For Realist Synthesis (for researchers and peer-reviewers)*. 2014. http://www.ramesesproject.org/media/RS_qual_standards_researchers.pdf (accessed 7 Dec 2017).

84 Wong G, Greenhalgh T, Westhorp G, *et al.* RAMESES publication standards: realist syntheses. *BMC Med* 2013;**11**:21. doi:10.1186/1741-7015-11-21

85 Booth A. Systematic searching for theory to inform systematic reviews: is it feasible? Is it desirable? *Health Info Libr J* 2015;**32**:220–35.http://ovidsp.ovid.com/ovidweb.cgi?T=JS&PAGE=reference&D=emed17&NEWS=N&AN=607682447 (accessed 7 Dec 2017).

For peer review only

86 Booth A, Harris J, Croot E, *et al.* Towards a methodology for cluster searching to provide conceptual and contextual 'richness'; for systematic reviews of complex interventions: case study (CLUSTER). *BMC Med Res Methodol* 2013;**13**. doi:10.1186/1471-2288-13-118

87 Greenhalgh T, Peacock R. Effectiveness and Efficiency of Search Methods in Systematic Reviews of Complex Evidence: Audit of Primary Sources. *BMJ* 2005;**331**:1064–5. doi:10.1136/bmj.38636.593461.68

88 Pawson R, Tilley N. How to Design a Realistic Evaluation. In: *Realistic Evaluation*. London: : SAGE Publications Ltd 1997. 83–114.

89 Wong G, Westhorp G, Pawson R, *et al. Realist Synthesis: RAMESES Training Materials*. 2013. http://www.ramesesproject.org/media/Realist_reviews_training_materials.pdf (accessed 7 Dec 2017).

90 Alger I, Salanié F. A theory of fraud and overtreatment in experts markets. *J Econ Manag Strateg* 2006;**15**:853–81. doi:10.1111/j.1530-9134.2006.00120.x

91 Hozo I, Djulbegovic B. When Is Diagnostic Testing Inappropriate or Irrational? Acceptable Regret Approach. *Med Decis Mak* 2008;**28**:540–53. doi:10.1177/0272989X08315249

92 Djulbegovic B, van den Ende J, Hamm RM, *et al.* When is rational to order a diagnostic test, or prescribe treatment: The threshold model as an explanation of practice variation. *Eur J Clin Invest* 2015;**45**:485–93. doi:10.1111/eci.12421

93 Hozo I, Tsalatsanis A, Djulbegovic B. Expected utility versus expected regret theory versions of decision curve analysis do generate different results when treatment effects are taken into account. *J Eval Clin Pract* Published Online First: 2016. doi:10.1111/jep.12676

94 Woodward RS, Schnitzler MA, Kvols LK. Reduced uncertainty as a diagnostic benefit: An initial assessment of somatostatic receptor scintigraphy's value in detecting distant metastases of carcinoid liver tumours. *Health Econ* 1998;**7**:149–60.

95 Croskerry P, Petrie DA, Reilly JB, *et al.* Deciding about fast and slow decisions. *Acad Med* 2014;**89**:197–200. doi:10.1097/ACM.0000000000000121

96 Aspinall P, Hill AR. Clinical Inferences and Decisions-II. Decision Trees, Receiver Operator Curves and Subjective Probability. *Ophthalmic Physiol Opt* 1984;**4**:31–8. doi:10.1111/j.1475-1313.1984.tb00329.x

97 Nilsen P, Roback K, Broström A, *et al.* Creatures of habit: accounting for the role of habit in implementation research on clinical behaviour change. *Implement Sci* 2012;**7**.https://implementationscience.biomedcentral.com/articles/10.1186/1748-5908-7-53 (accessed 21 Dec 2017).

98 Zaat JOM, van Eijk JTM, Bonte HA. Laboratory Test Form Design Influences Test Ordering by General Practitioners in the Netherlands. *Med Care* 1992;**30**:189–98.http://www.jstor.org/stable/3766214 (accessed 21 Dec 2017).

99 Van Bokhoven MA, Koch H, Van Der Weijden T, *et al.* The effect of watchful waiting compared to immediate test ordering instructions on general practitioners' blood test ordering behaviour for patients with unexplained complaints; a randomized clinical trial (ISRCTN55755886). *Implement Sci* 2012;**7**. doi:10.1186/1748-5908-7-29

100 Verstappen WHJM, Van Der Weijden T, Dubois WI, *et al.* Improving test ordering in primary care: The added value of a small-group quality improvement strategy compared with classic feedback only. *Ann Fam Med* 2004;**2**:569–75. doi:10.1370/afm.244

101 Morgan S, Saltis T, Coleman J, *et al.* Test result audit and feedback (TRAFk) as a supervision method for rational test ordering in general practice training. *Aust Fam Physician* 2016;**45**:518–22.https://www.racgp.org.au/download/Documents/AFP/2016/July/AFP-July-Research-Morgan.pdf (accessed 21 Dec 2017).

102 Trietsch J, van Steenkiste B, Grol R, *et al.* Effect of audit and feedback with peer review

For peer review only

on general practitioners' prescribing and test ordering performance: a cluster-randomized controlled trial. *BMC Fam Pract* 2017;**18**. doi:10.1186/s12875-017-0605-5
103 Poley MJ, Edelenbos KI, Mosseveld M, *et al.* Cost Consequences of Implementing an Electronic Decision Support System for Ordering Laboratory Tests in Primary Care: Evidence from a Controlled Prospective Study in The Netherlands. *Clin Chem* 2007;**53**:213–9. doi:10.1373/clinchem.2006.073908
104 Martins CMS, da Costa Teixeira AS, de Azevedo LFR, *et al.* The effect of a test ordering software intervention on the prescription of unnecessary laboratory tests - a randomized controlled trial. *BMC Med Inform Decis Mak* 2017;**17**:20. doi:10.1186/s12911-017-0416-6
105 Wong G, Greenhalgh T, Westhorp G, *et al.* Realist methods in medical education research: What are they and what can they contribute? *Med Educ* 2012;**46**:89–96. doi:10.1111/j.1365-2923.2011.04045.x
106 Moher D, Liberati A, Tetzlaff J, *et al.* Preferred Reporting Items for Systematic Reviews and Meta-Analyses: The PRISMA Statement. *PLoS Med* 2009;**6**:e1000097. doi:10.1371/journal.pmed.1000097
107 Pawson R. Digging for Nuggets: How 'Bad' Research Can Yield 'Good' Evidence. *Int J Soc Res Methodlogy* 2006;**9**:1364–5579. doi:10.1080/13645570600595314
108 Wong G, Brennan N, Mattick K, *et al.* Interventions to improve antimicrobial prescribing of doctors in training: the IMPACT (IMProving Antimicrobial presCribing of doctors in Training) realist review. *BMJ Open* 2015;**5**:e009059. doi:10.1136/bmjopen-2015-009059
109 RAMESES II Project. Retroduction in realist evaluation. 2017.http://www.ramesesproject.org/media/RAMESES_II_Retroduction.pdf (accessed 22 Nov 2017).
110 Preventing Overdiagnosis. http://www.preventingoverdiagnosis.net
111 International Realist Conference. http://realist2017.org

Figure legends

Figure 1: Steps to over/underdiagnosis
Figure 2: Review project design
Figure 3: Contexts, reasons for test-ordering and range of outcomes
Figure 4: Example CMOCs showing the possible effect of introducing reviews of test ordering behaviour

For peer review only

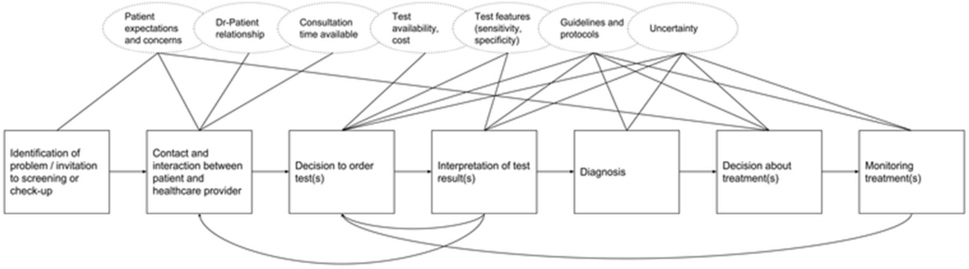

Figure 1: Steps to over/underdiagnosis

33x10mm (600 x 600 DPI)

peer review only

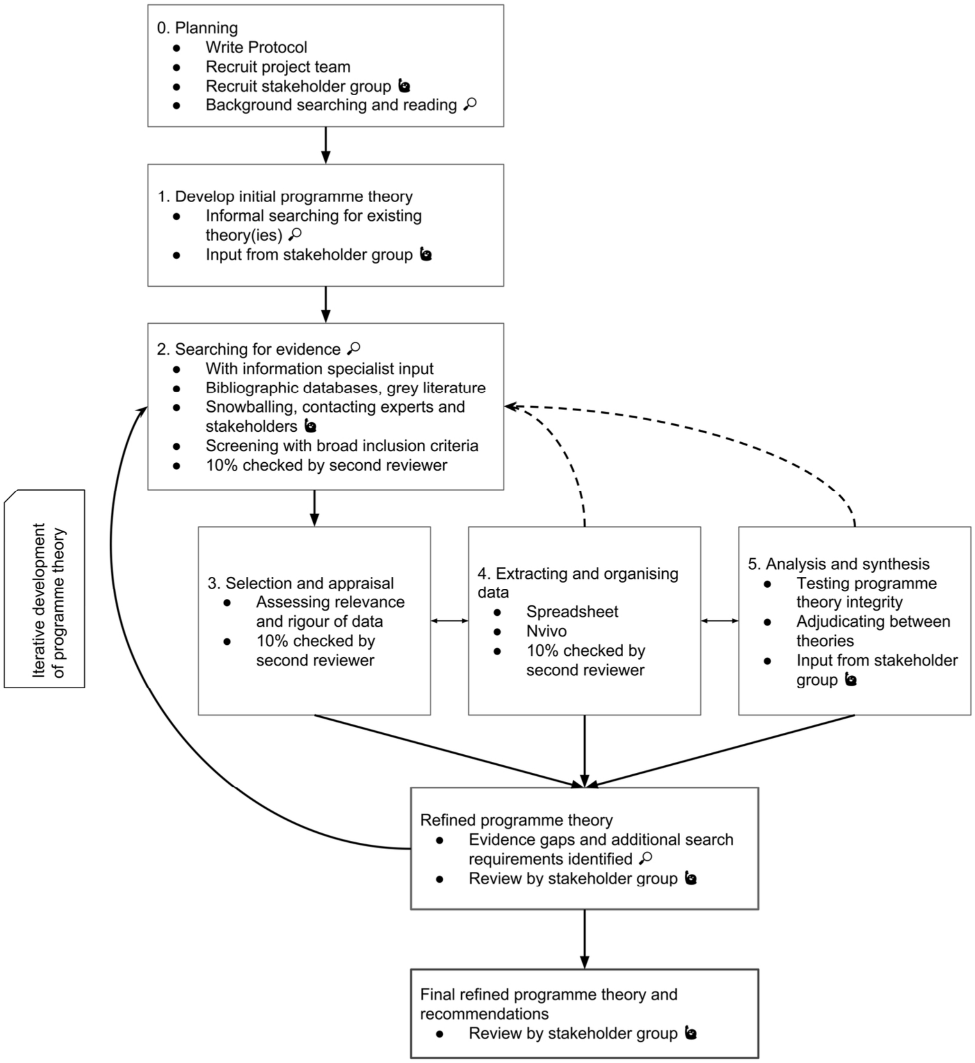

Figure 2: Review project design

70x76mm (600 x 600 DPI)

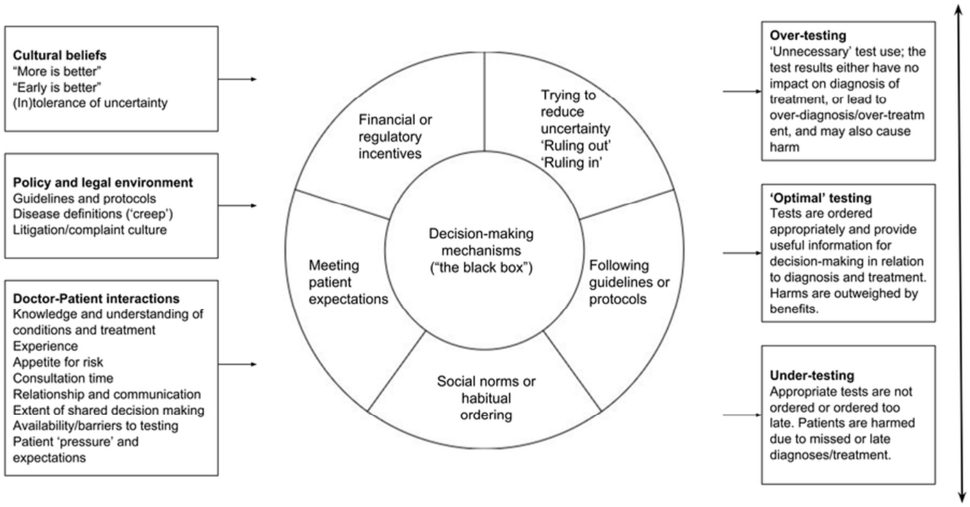

Figure 3: Contexts, reasons for test-ordering and range of outcomes

39x20mm (600 x 600 DPI)

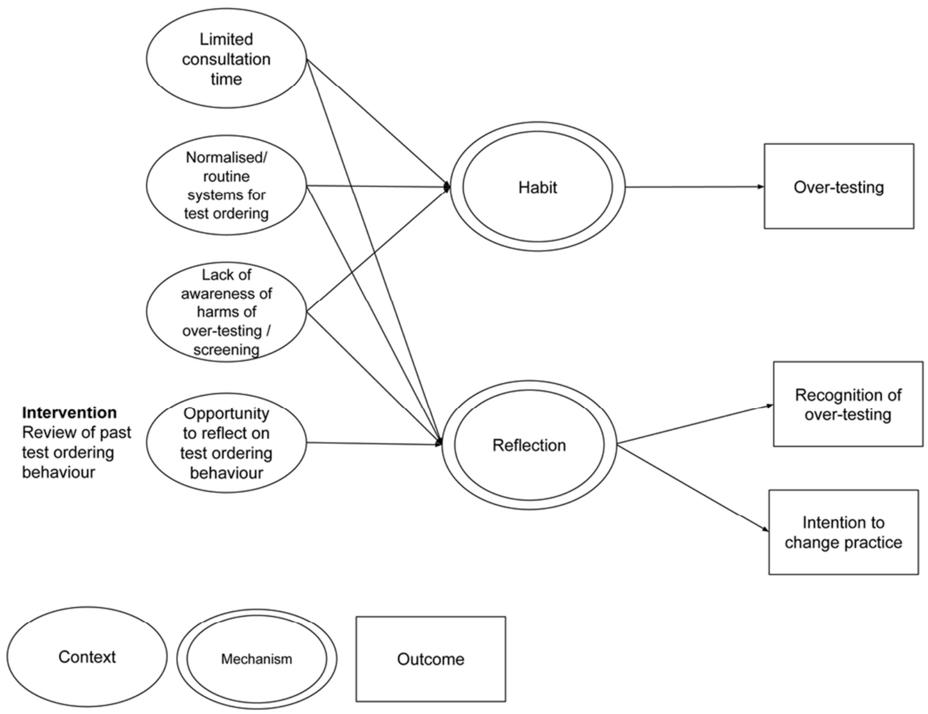

Figure 4: Example CMOCs showing the possible effect of introducing reviews of test ordering behaviour

47x35mm (600 x 600 DPI)

Growth in 'overdiagnosis' literature

This chart shows the dramatic increase in published literature on the topic of 'overdiagnosis' since the late 2000s as awareness of the problem has grown.

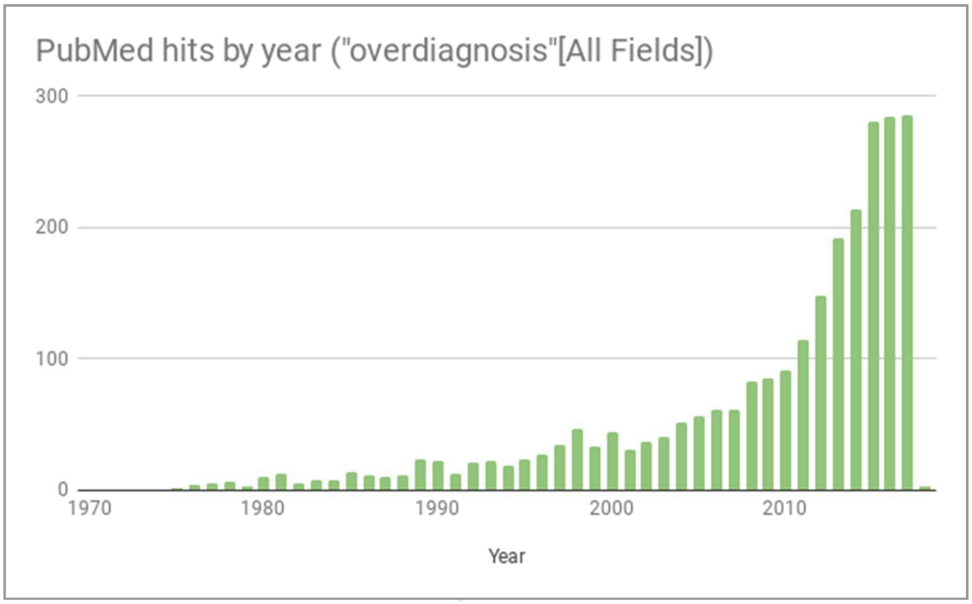

A specific MeSH term, "Medical Overuse", covering overdiagnosis, overtreatment and related 'too much medicine' concepts was introduced in 2016, another sign of increasing interest (previously articles on this topic were indexed as part of the broader "Unnecessary Procedures" category (introduced in 1997).

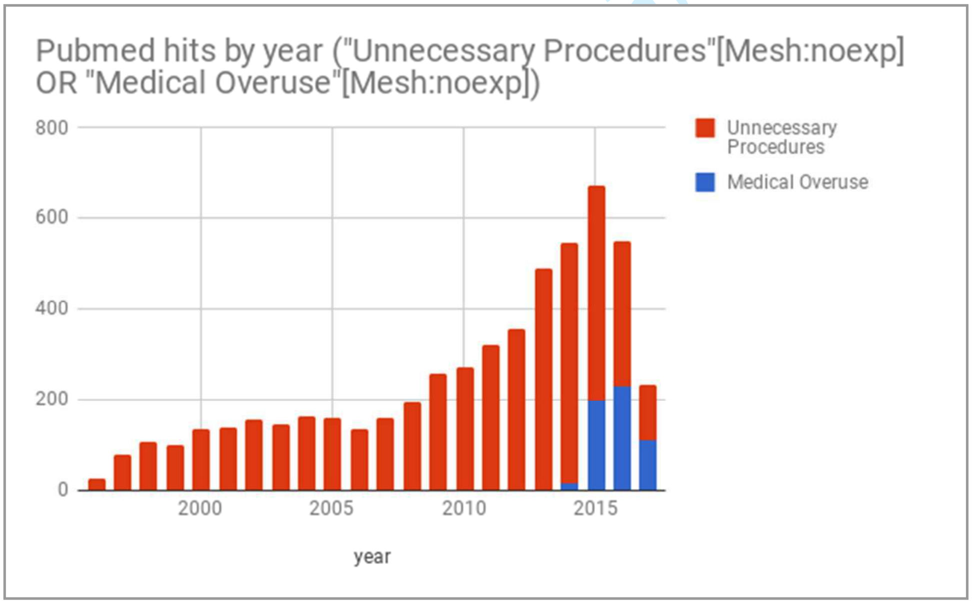

Glossary of realist terminology
(adapted from Realist Synthesis: RAMESES Training Materials)[1]

CMOC	"Context-mechanism-outcome configuration"; a diagrammatic or narrative description offering an explanation of the relationship between some particular context(s), mechanism(s) and outcome(s).<br><br>Multiple CMOCs may exist within a single programme theory.
Context	"Any condition that triggers and/or modifies the behaviour of a mechanism"[2]<br><br>Context refers to the important feature(s) of the circumstances in which a programme exists (or a phenomenon happens) which 'trigger' the mechanisms that generate outcomes. Changes in context over time or in different settings will affect whether and which mechanisms are in operation.
Demi-regularity	A semi-predictable pattern, such as we expect to observe in outcomes that depend on human choice or agency.[2]
Folk theory	An informal theory describing how an intervention is assumed or known to work, or how a phenomenon is assumed or known to come about, according to those practitioners or participants directly involved
Mechanism (generative)	The underlying process by which outcomes are generated. Mechanisms are usually descriptions of the tendencies, reasoning and behaviour of agents involved in a process or participants in a programme and their response to the important context(s) in which they exist.<br><br>Mechanisms are distinguished from "variables or correlates"[3] that are associated with particular outcomes; instead they offer an explanation for why and how observed outcomes happen.
Middle-range theory	A theory that is specific enough to be tested (e.g. against secondary evidence in a realist review, or against primary evidence in a realist evaluation), but abstract or generalisable enough to be transferable, and have explanatory value in other situations.
Programme theory	A theory that describes what an intervention comprises and how it is expected to work, or the process by which the outcomes of interest are thought to come about (expressed as a narrative description or in a diagram).<br><br>A realist programme theory is expressed in terms of the relationship between relevant context(s), mechanism(s) and outcome(s) (or CMOCs).
Substantive theory	An existing established theory from any discipline that can be used to help understand the intervention or phenomenon under examination.

For peer review only

Strategies for early theory-seeking searches

PubMed (19/12/2017)

1	((overdiagnos* OR over-diagnos* OR overtreat* OR over-treat* OR over-prescri* OR overprescri* OR "Unnecessary Procedures"[Mesh])) AND ((("logic model" OR "theory of change" OR "theory of action" OR "outcomes chain" OR "program* theory" OR "program* logic" OR "logical framework*")))	16 hits
2	(overtest* OR over-test* OR “inappropriate test*” OR “unnecessary test*” OR “irrational test*”) AND ("logic model" OR "theory of change" OR "theory of action" OR "outcomes chain" OR "program* theory" OR "program* logic" OR "logical framework*")	5 hits
3	((overdiagnos* OR over-diagnos* OR overtreat* OR over-treat* OR over-prescri* OR overprescri* OR "Unnecessary Procedures"[Mesh])) AND (("theor*" OR "framework*" OR "concept*" OR "model*"))	999 hits
4	(overtest* OR over-test* OR “inappropriate test*” OR “unnecessary test*” OR “irrational test*”) AND (theor* OR framework* OR concept* OR model*)	154 hits

Google Scholar (19/12/2017)

(overdiagnosis OR Overtreatment OR overtesting OR "unnecessary test" OR "inappropriate test" OR "irrational test") AND (theory OR framework OR concept* OR model*)
42,100 hits (first 15 pages screened)

(overdiagnosis OR overtreatment OR overtesting) AND ("logic model" OR "theory of change" OR "theory of action" OR "outcomes chain" OR "program theory" OR "program logic" OR "logical framework")
164 hits

References

1 Wong G, Westhorp G, Pawson R, *et al. Realist Synthesis: RAMESES Training Materials*. 2013. http://www.ramesesproject.org/media/Realist_reviews_training_materials.pdf (accessed 7 Dec 2017).
2 Jagosh J, Macaulay AC, Pluye P, *et al.* Uncovering the Benefits of Participatory Research: Implications of a Realist Review for Health Research and Practice. *Milbank Q* 2012;**90**:311–46. doi:10.1111/j.1468-0009.2012.00665.x
3 Pawson R, Tilley N. *Realistic evaluation*. London: : Sage 1997.

For peer review only

BMJ Open

Explaining variations in test ordering in primary care: protocol for a realist review.

Journal:	*BMJ Open*
Manuscript ID	bmjopen-2018-023117.R1
Article Type:	Protocol
Date Submitted by the Author:	29-Jun-2018
Complete List of Authors:	Duddy, Claire; University of Oxford, Nuffield Department of Primary Care Health Sciences<br>Wong, Geoffrey; University of Oxford, Nuffield Department of Primary Care Health Sciences
**Primary Subject Heading**:	Pathology
Secondary Subject Heading:	Health services research
Keywords:	PRIMARY CARE, PATHOLOGY, Quality in health care < HEALTH SERVICES ADMINISTRATION & MANAGEMENT

SCHOLARONE™
Manuscripts

For peer review only - http://bmjopen.bmj.com/site/about/guidelines.xhtml

Explaining variations in test ordering in primary care: protocol for a realist review.

Duddy, Claire* and Wong, Geoff

***Corresponding author**
Claire Duddy

Nuffield Department of Primary Care Health Sciences
Radcliffe Observatory Quarter
Woodstock Road
Oxford OX2 6GG

claire.duddy@phc.ox.ac.uk
+44 (0)1865 289300

Other authors
Geoff Wong
Nuffield Department of Primary Care Health Sciences, University of Oxford, Oxford, UK

Word count (excluding title page, abstract, references, figures, tables): 4724

For peer review only

ABSTRACT

Introduction

Studies have demonstrated the existence of significant variation in test ordering patterns in both primary and secondary care, for a wide variety of tests and across many health systems. Inconsistent practice could be explained by differing degrees of under- and overuse of tests for diagnosis or monitoring. Underuse of appropriate tests may result in delayed or missed diagnoses; overuse may be an early step that can trigger a cascade of unnecessary intervention, as well as being a source of harm in itself.

Methods and analysis

This realist review will seek to improve our understanding of how and why variation in laboratory test ordering comes about. A realist review is a theory-driven systematic review informed by a realist philosophy of science, seeking to produce useful theory that explains observed outcomes, in terms of relationships between important contexts and generative mechanisms.

An initial explanatory theory will be developed in consultation with a stakeholder group and this ‘programme theory’ will be tested and refined against available secondary evidence, gathered via an iterative and purposive search process. This data will be analysed and synthesised according to realist principles, to produce a refined ‘programme theory’, explaining the contexts in which primary care doctors fail to order ‘necessary’ tests, and/or order ‘unnecessary’ tests, and the mechanisms underlying these decisions.

Ethics and dissemination

Ethical approval is not required for this review. A complete and transparent report will be produced in line with the RAMESES standards. The theory developed will be used to inform recommendations for the development of interventions designed to minimise ‘inappropriate’ testing. Our dissemination strategy will be informed by our stakeholders. A variety of outputs will be tailored to ensure relevance to policy makers, primary care and pathology practitioners, and patients.

Keywords: realist review, variation, test ordering, primary care

For peer review only

Strengths and limitations of this study

- First realist review exploring how, why and in what circumstances variations in test ordering in primary care come about;
- Realist approach embraces complexity, seeking to develop understanding of multiple causes of variation and to explore the role of different contexts;
- Involvement of stakeholders in refining programme theory and disseminating outputs will ensure relevance and applicability;
- Availability and richness of available evidence may limit theory building.

BACKGROUND

Variation in test ordering

A large number of studies and reports have demonstrated the existence of significant variation in primary and secondary care test ordering patterns, across many different health systems.[1–15] This variation in practice could be explained by differing degrees of under- and overuse of diagnostic testing in these different settings. Primary studies and reviews that attempt to assess the extent of 'inappropriate' test use usually assess observed test use against chosen guideline standards.[16,17] This approach has limitations, as assessments can only be made wherever guidelines or protocols exist, and will only be as reliable as the guidelines themselves.

This review is concerned with the use of laboratory tests in primary care settings. Our initial focus will be on the NHS in the UK, but we will endeavour to develop recommendations relevant in other settings and countries, where it is likely that the same mechanisms and contexts produce similar outcomes. The use of such tests in UK primary care is extensive, and growing,[15] and is known to vary substantially by region.[13,15] In 2006, the Carter Review reported that 35-45% of requests for laboratory tests in the UK came from primary care.[18] Although an individual laboratory test may be inexpensive, high volumes mean that overall expenditure is high. The same review estimated that pathology services cost the NHS around £2.5 billion per year.[18]

Undertesting and overtesting

Although variations in test ordering practice clearly occur, categorising this practice as under- or overtesting can be more difficult. As noted above, existing studies usually rely on assessing test ordering behaviour against existing guideline or protocol standards. For individual patients, it may only be possible to decide that a particular testing decision was 'inappropriate' later, in light of the results and subsequent decisions (and in many cases, this may be impossible to ascertain even then.[19,20] The picture is further complicated by the possibility that under- and overtesting may occur simultaneously.[21]

It is clear however that both under- and overtesting can have negative consequences for patients. Underutilisation of appropriate tests can result in delayed, missed or incorrect diagnoses and subsequent treatment, and failure to appropriately monitor patients with existing conditions can also result in harm. Uneven access to tests and treatment for different population groups is also a concern.[22–24]

For peer review only

Overtesting is also a problem. Overdiagnosis and consequent overtreatment are increasingly seen as an important source of harm within many healthcare systems. The phenomenon of 'too much medicine' is considered by many to result in direct and indirect harm to individual patients in the form of unnecessary labelling and treatment[25–28] as well as posing a threat to sustainability and equity in healthcare systems, increasing costs[29,30] and diverting resources from the genuinely ill to the 'worried well'.[31]

The increasing interest in this area is reflected in campaigning, including the BMJ's 'Too Much Medicine'[32] (launched in 2002) and 'Choosing Wisely'[33] (launched in the UK in 2016), in a growing number of popular books[34–37] and articles in the mainstream media,[38–41] and in a rapidly growing literature (see Supplementary File). A recent wide-ranging (though not systematic) review[42] drew attention to the large number of 'drivers' of medical overuse that have been identified, but also highlighted the limitations of the existing literature, which is dominated by "analyses or commentaries".[42]

Medical overuse, including overtesting, is often considered under the 'overdiagnosis' banner. Precise definitions are contested,[19,43,44] but terminology like 'overdiagnosis' is frequently used broadly by both researchers and activists to cover a wide range of issues. A broad conceptualisation encompasses concerns ranging from the over-detection of harmless cancers during screening (and their subsequent overtreatment)[45] to widening definitions of disease and pre-disease,[28,46] and many more. The common thread is the identification of medical care that is provided despite "a low probability of benefiting the person diagnosed"[47] and indeed, the possibility that such care may instead be a source of harm.

'Overtesting' may therefore be defined in these terms, as the use of tests where there is a low probability that test results will benefit the patient. This could be the case where there is a lack of evidence to support the use of a test, the use of tests where their results are unlikely to change subsequent management or unnecessary repeat test ordering. Conversely, 'undertesting' may occur in the opposite circumstances.

Overdiagnosis and overtreatment phenomena are usually quantified only at population level.[44,48] However, outcomes of under- and overtesting are the cumulative effect of many individual decisions taken in a variety of circumstances, within the social system of healthcare. A preliminary map of the decisions faced by both patients and doctors in a primary care setting, alongside some important contextual considerations, is provided below in Figure 1.

[Figure 1 here]

The decision to order tests is an important feature of this process and an over-reliance on testing has been identified as an important early step that may result in a cascade of further testing and intervention, including the potential for overdiagnosis and overtreatment.[35,49,50] In addition, overtesting and its consequences can directly increase anxiety and worry for

For peer review only

patients[51–53] and commentators have highlighted the limited capacity of even ‘gold standard’ tests in providing definitive diagnostic answers.[44]

Existing reviews

Two existing systematic reviews assess ‘inappropriate’ under- and overtesting in secondary[14] and primary[12] care settings: both identified significant variation in practice across a wide range of tests and settings. One health technology assessment considers the extent and consequences of routine pre-operative testing.[54] In addition, several systematic reviews assess the efficacy of various interventions designed to reduce variability and improve ‘appropriateness’ of test ordering in a wide variety of settings.[55–67] One review considers a wide range of variables associated with ‘test-ordering tendencies’.[68]

No realist reviews on this subject have been found. The wide variation in test-ordering behaviour, and in the outcomes of studies aiming to reduce ‘inappropriate’ testing indicates that an enquiry into the role of context could have explanatory value for this phenomenon. Patterns of test-ordering behaviour may vary in response to important contextual factors, such as those highlighted in Figure 1 above. A number of existing studies have highlighted the wide variety of potential drivers of variation in practice, including clinician and patient characteristics[68,69] and health system characteristics.[2,6,11,68,70] A realist review of the literature will allow consideration of multiple causal mechanisms, sensitivity to context and opening the ‘black box’[71] of decision-making in relation to ordering tests.

Realist review

A realist review (otherwise known as ‘realist synthesis’) is an interpretive, theory-driven systematic literature review,underpinned by a realist philosophy of science. This philosophy holds that patterns of observed (empirical) outcomes are produced by underlying ‘generative’ (real) mechanisms, which may or may not be at work in particular contexts.[72] ‘Mechanisms’ are understood as the causal forces of patterns of observed outcomes (or ‘demi-regularities’) that have their roots in individual tendencies and reasoning.[73] Causation is ‘generative’, that is, outcomes in social systems are not the direct result of interventions or simple responses to stimuli, but rather reflect the invisible reasoning and behaviour of actors within those systems.[74] Such reasoning may change (or not) in different contexts, where different resources are available to different actors with different capacities to respond to their circumstances. The realist approach can allow us to go beyond an assessment of those variables associated with a particular outcome, to shed light on the real generative mechanisms that are the underlying causes of observed test-use, and to highlight the context(s) or conditions in which these mechanisms operate.[75] Contexts and mechanisms are seen as working together to produce outcomes (often expressed as, $C + M \rightarrow O$).[76]

A realist approach may be adopted when there is a need to account for inconsistent outcomes and differences in context, to understand underlying causation and to answer questions that begin ‘how’, ‘why’, ‘in what circumstances’, ‘for whom’ and so on.[77] Originally proposed as a means to explore the inner workings of similar ‘families’ of complex social interventions,[73] its

For peer review only

utility in helping to 'diagnose' and understand the underlying nature of complex problems has also been established.[78,79] For a glossary of realist terminology, see Supplementary File.

Here, the overall problem of medical overuse and the specific issues of over- and undertesting are characterised as 'complex': the literature suggests multiple potential causes operating at different levels, as well as potential emergent effects, whereby (for example) more testing begets even more testing,[25,80] and variable outcomes exist (undertesting and overtesting coexist in the same healthcare system, for example).[12,81] Decisions to order tests in primary care are made within the context of the interaction between provider and patient; as such there are multiple opportunities for the reasoning and behaviour of both parties to influence the outcome.[82]

Realist inquiry begins (and ends) with a 'programme theory', describing a hypothesis about how an intervention works or how a phenomenon comes about. Realist programme theories are models that describe relationships between important contexts, mechanisms and outcomes, usually presented and described as sequences of 'context-mechanism-outcome configurations' ('CMOCs'). Such configurations aim to explain in which context(s), which mechanism(s) are 'triggered' to produce which outcomes(s). As such, the realist approach is especially useful where outcomes appear to vary with circumstances, seeking to provide explanatory evidence for such variation, and offers a means of adjudicating between competing theories and/or refining and improving an initial theory to accommodate multiple explanatory mechanisms.[75]

A realist programme theory should be in the 'middle range', i.e. it should be specific enough to permit empirical testing (in this case, against secondary evidence located during the review process), but abstract enough to provide useful, explanatory transferability to other situations where the same mechanisms may be operating.[83]

REVIEW OBJECTIVES AND DESIGN

Review Objectives

1. Develop a realist programme theory offering explanation(s) for the variation in test ordering in primary care, underpinned by secondary evidence.
2. Make recommendations based upon this explanation, to inform the design of existing and new interventions that could help to reduce this problem.

Review Questions

1. How are 'undertesting' and 'overtesting' conceptualised in the literature?
2. In what contexts do primary care doctors order 'unnecessary' tests?
3. In what contexts do primary care doctors fail to order 'necessary' tests?
4. What mechanisms are at work in these different contexts that underlie test-ordering behaviour and generate these outcomes?

The review will be conducted according to Pawson's five stages[84,85] which outline the processes by which an initial programme theory will be developed, evidence gathered and refinements to theory made. The RAMESES quality[86] and reporting[87] standards will be

For peer review only

followed. Figure 2 summarises the overall project design, and more details on each step are provided below.

A “guiding principle” of the realist approach is the maintenance of transparency of methods and decision-making throughout the review.[87] Such transparency ensures that the iterative nature of the research is made clear and that decisions taken in consultation with stakeholders and within the project team are fully explained and justified. Such decisions determine the direction and focus of the project, as well as guiding the extent and direction of literature searching, and the analysis and synthesis themselves.

[Figure 2 here]

Stakeholder involvement

Following an established approach,[79] a diverse stakeholder group will be recruited at the beginning of the project. This group will include, for example, primary care clinicians, pathologists, managers and policymakers. The involvement of stakeholders at multiple stages is made clear in Figure 2. This group will provide the content expertise essential for initial programme theory development and beyond. We will consult this group when focusing our review question and in assessing and developing candidate programme theories, to check that stakeholders agree that the theories under consideration are relevant, and resonate with their experience.[86] Stakeholders may also suggest useful sources of evidence, and members of the group will be asked to provide feedback on iterations of refined programme theory as these are developed. Finally, the stakeholder group will be crucial in helping us to identify the most effective means of disseminating the results and recommendations that follow from the review.

Patient and public involvement

Patients and the public will be involved throughout this review project via their inclusion as part of our stakeholder group. This means that patients will have the opportunity to help us prioritise the focus of this review and to develop and ‘test’ our programme theories as they develop. In particular we anticipate that patient input (as well as input from clinicians) will help us to identify and understand the important contexts, and reasoning at work whenever there is a decision to order tests (or otherwise). This input will help inform our searching, and development of theory and ensure that the final refined programme theory resonates with patient experience.

Step 1: Develop initial programme theory

The first stage of a realist review is the development of an ‘initial programme theory’ which makes a first attempt to explain the phenomenon under examination. The development of this theory will be informed by two main processes: an informal scoping search of the literature, and input from the stakeholder group.

Iterative, informal searching will be used to locate existing theories that are used to explain how and why overtesting and undertesting occur. This initial search stage will rely on a combination of more structured searching[88,89] and more emergent techniques such as reference and citation tracking (‘snowballing’) and personal contacts.[90] An inclusive approach will be used to

For peer review only

screen documents found at this stage, with no limitations placed on type of study or document. Documents will be selected wherever there is an attempt to theorise about the causes of variation in test ordering, especially in relation to the circumstances in which such variation is most prevalent, and the reasoning of actors involved (even where such ideas are not identified formally as 'theory').

This process may uncover informal 'folk theories'[91] attempting to explain the causes of variations in practice, and theories that underpin actual and proposed interventions designed to reduce the problem,[73] as well as potentially useful 'substantive' theory,[92] i.e. established theory from any discipline which can help to explain the phenomenon. The stakeholder group will also be consulted to ensure that their content expertise is used to supplement the results of this early searching. Candidate initial programme theories will be presented, and stakeholders asked to provide feedback and commentary on their plausibility and 'fit' with their experience. Through this process, initial theory(ies) are likely to be refined and prioritised for the next stage of the review.

Work on this stage has begun and is ongoing. Initial search strategies focused on identifying relevant substantive theories are available in the Supplementary File. Figure 3 below illustrates the basis of an early set of initial programme theories, considering the 'decision to order test(s)' step from Figure 1 above.

[Figure 3 here]

Initial exploration of the literature has uncovered a range of potentially useful substantive theory that could help to explain the mechanisms underlying the decision-making involved in test-ordering behaviour, including economic theory explaining over-supply and over-consumption in "experts markets",[93] theories of decision-making that assume bounded rationality[94], including regret theory[95] and threshold models[96] and several others.[97–99] These theories can be explored in relation to their ability to provide a useful lens through which to view this decision-making process in a realist fashion and explain observed outcomes. For example, 'regret theory' suggests the possibility of an underlying mechanism related to the estimation and minimisation of 'expected regret' in deciding to order a test or otherwise.

Another potentially valuable source in the development of initial programme theory are those theories underlying interventions designed to reduce overtesting. Instead of assuming a complex decision-making process is happening, many such interventions seem based on the theory that test-ordering is at least to some extent a habitual, normalised behaviour[100] and so seek to disrupt these habits. For example, interventions designed to increase barriers to test-ordering[101,102] may create space for doctors to consider whether a test is really necessary. Similarly, interventions designed to promote reflective practice[103–105] provide opportunities for doctors to reflect on their past test-ordering behaviour and outcomes and potentially change their behaviour in the future. Interventions based around computer-aided decision-support systems[106,107] may seek to replace old habits with new, evidence-based ones.

For peer review only

These initial theories can be conceptualised in a 'realist' fashion (i.e. in the form of a CMOC), as illustrated in the hypothesised example in Figure 4 below.

[Figure 4 here]

The candidate theories uncovered during searching will be considered by the project team alongside Figure 3 to refine these initial CMOCs. These will be discussed with the stakeholder group and refined as necessary in light of these discussions and further reading. It is likely that a small number of candidate theories will be prioritised as a focus for the review, based on their greater importance and/or resonance with stakeholders.

Step 2: Searching for evidence

Secondary evidence gathered in cycles over the course of a realist review is iteratively interpreted and used to "confirm, refute or refine" each aspect of a programme theory.[108] This evidence is sought from a wide range of sources and disciplines: there is no 'hierarchy of evidence' in a realist approach and so evidence may include quantitative and qualitative data, peer-reviewed articles, opinion and commentary, and grey literature like policy documents.[92]

The main systematic literature search(es) will be conducted with the aim of identifying relevant documents potentially containing data that can be used to develop or refine, refute or confirm, the initial programme theory(ies) chosen for testing.

A search strategy(ies) will be designed, piloted and executed by an information specialist (CD). A wide range of bibliographic databases covering multiple disciplines will be considered for searching, including MEDLINE, Embase, CINAHL, PsycINFO, PsycEXTRA, the Web of Science Core Collection, Scopus, ASSIA, IBSS, EconLit and Google Scholar. Sources of grey literature will be searched, including via web search engines. Free text and subject heading search terms will be chosen as appropriate, and the search strategy will be refined iteratively to achieve a balance of sensitivity and specificity. As for the informal search stage, 'snowballing' and other supplementary search techniques will be used to identify additional documents.[90]

Search results will be screened initially by title and abstract, with full text considered as a second step. A broad set of inclusion and exclusion criteria will be used to screen the results of the main search. These criteria will be finalised when the initial programme theory is confirmed, but are likely to include some or all of the following:

Inclusion criteria

- All types of document;
- Any study design;
- Studies or documents that identify variation in test use, actual or potential under- or overuse of tests, or are focused on areas of primary health care where under- or overtesting is a recognised problem;
- Studies or documents focused on primary care settings;

For peer review only

- If a particular type of test or specific test is chosen as a focus in consultation with the stakeholder group, searching may initially be limited to consider this area.

Exclusion criteria

- Studies or documents focused on secondary care settings (though searches may be broadened later to consider additional settings if there is a dearth of literature focused on primary care, or where the stakeholder group or initial searches suggest common mechanisms may be in operation);
- Studies focused on imaging, genetic testing, foetal monitoring, near-patient testing, self-testing, or home-based testing by patients (though searches may be broadened later, as above);
- Studies or documents focused on low and middle income settings, where limited resources are likely to create very different contextual factors that are outwith the scope of this review.

Screening of titles and abstracts will be undertaken primarily by the first reviewer (CD). An initial pilot batch of documents will be screened in duplicate by GW and the review team will meet to discuss discrepancies and assess agreement between the reviewers. Thereafter a 10% random sample of search results will be screened by the second reviewer (GW) to check for consistency. Disagreements will be recorded and resolved via discussion in the project team.

As Figure 2 illustrates, additional searching may be undertaken as required at later stages of the review, wherever the main search did not generate sufficient data to test programme theory (e.g. if data on particular contexts or mechanisms was sparse), or in response to potential programme theory refinements. All such additional searches will be developed with an information specialist and screened as described above.

All searching and screening processes will be reported in full, including PRISMA-style flow diagrams,[109] to ensure transparency of evidence sources.

Step 3: Selection and appraisal

Following screening, documents will be selected on the basis of an assessment of their relevance (i.e. whether some part(s) of the document can contribute to the refinement of programme theory) and rigour (i.e. the trustworthiness of that data).[85] One reviewer (CD) will read all of the documents that met the inclusion criteria during screening and assess their ability to speak to some aspect of the programme theory under consideration (i.e. relevance). Relevant data from these documents will then be assessed for rigour.

The assessment of rigour in a realist review is not conducted at article- or document-level as in a 'traditional' review, since doing so may exclude documents containing relevant data[92] and even where a study as a whole is methodologically weak in terms of its own objectives, it may still contain 'nuggets' of useful data.[110] Instead, each piece of relevant contributing data will be judged according to its purpose in testing programme theory[85] and the methodology by which the particular piece of data was produced. This may involve the use of formal critical appraisal

For peer review only

checklists suitable for different study types, but only as one part of determining trustworthiness. Different types of data will be subject to different judgements of methodological coherence and plausibility,[92] and the details of each assessment will be recorded in full to ensure that this process is transparent.

As with screening, a 10% random subsample of documents will be assessed by a second reviewer (GW) using the same criteria, with disagreements recorded and resolved via discussion in the project team. It anticipation of uncertainty in the case of some documents, the project team may also be called upon to make assessments as a group.[79,111]

Step 4: Extracting and organising data

One reviewer will extract the main characteristics of each included document into an Excel spreadsheet. The full text of all of the documents will then be uploaded into the NVivo QRS International qualitative data analysis tool. One reviewer will then organise and classify this data, by annotating (coding) relevant data from each document according to its contribution to the developing programme theory.[85]

The initial phase of organising and coding data will be informed by any contexts, mechanisms and outcomes (or concepts not yet clarified as C, M or O) identified in the development of the initial programme theory. As data extraction progresses, organisation and coding is likely to evolve and include new concepts that reflect refinements to programme theory. As such, each document may be subject to several readings. As noted above, an individual document may include sections that contribute to several elements of programme theory. The use of data to refine programme theory will be recorded, to enable transparent reporting and the inclusion of relevant document extracts within the synthesis.[85] A 10% random subsample of documents that have been through the data extraction and organising process will be reviewed by a second reviewer (GW) to check for consistency, with disagreements recorded and resolved via discussion in the project team.

Step 5: Analysis and synthesis

In a realist review, analysis and synthesis of the selected data proceed in parallel, and will begin at the same point as document selection and appraisal for relevance and rigour, and data extraction and organisation.[75] All three stages may thus proceed simultaneously (see Figure 2), as data are chosen, assessed, annotated and organised according to its potential role in refining the developing programme theory.

This process will be iterative[75]: the programme theory will be refined in stages as more and more data are considered. The stakeholder group will be consulted at various points to obtain feedback on the focus and development of the programme theory and the project timeline will permit pauses in analysis and synthesis for this purpose, and to allow further searching to be undertaken where gaps in the available secondary evidence are found.

Pawson suggests that realist analysis and synthesis should be a process of “juxtaposing, adjudicating, reconciling, consolidating and situating the evidence” in an effort to refine

For peer review only

programme theory.[85] As such, data relating to different aspects of the programme theory will be collected together and considered alongside each other, such that an assessment of the strength of evidence supporting the arguments that underpin each aspect of that theory can be made. A process of retroductive reasoning will then be applied, so that refinements to programme theory are made on the basis of what can plausibly be inferred by all the data available. Retroductive reasoning will be used to build explanatory realist theory(ies). This involves an interpretive process of considering which underlying causal mechanisms must be at work to deliver the observed patterns out of outcomes. The approach involves moving back and forth between concrete observations and theory-building, and hence between inductive and deductive reasoning.[112]

LIMITATIONS AND RISKS

An important potential limitation of this study will be the availability and contextual richness of the secondary evidence that is available.[75] Although initial scoping searches suggest that a significant amount of material on the subject of laboratory test-ordering does exist, it is possible that this material will not describe contextual factors in great detail or include enough relevant information on which to build theory. We will attempt to address this problem by ensuring that comprehensive and wide-ranging searching is undertaken by an information professional, that supporting and related information for all included studies is located wherever it exists,[89] and by contacting authors to ask for further detail as required.

In addition, there are important limitations that are inherent to the nature of the realist review. In particular, there is a limit to how much ground a single review can cover and so this review will necessarily prioritise certain elements of the process within which test ordering takes place[75] and will inevitably have to set aside some potentially important factors for future research. The final output of the review will be a (refined) theory that attempts to illuminate important contextual factors and underlying mechanisms; it is important to acknowledge that such theory can only ever represent partial knowledge that will be open to further refinement or refutation in the future.

OUTPUTS AND DISSEMINATION

A variety of project outputs are planned, to meet the needs of different groups, including national and local policy makers, leaders, employers and practitioners in primary care and pathology settings, and patients. To some extent, outputs will be guided by the review's conclusions and resulting recommendations that may have relevance in different contexts and at different levels.

The RAMESES reporting standards will be used to produce a complete and transparent report of this review – both for the funder and as a standalone publication.[87] The standalone publication will be for academic audiences and will be submitted as an article to a peer-reviewed journal. Other academic outputs will be prepared for presentation at relevant conferences (e.g. 'Preventing Overdiagnosis,[113] International Realist Conference.)[114]

For peer review only

The final refined programme theory and resulting recommendations will be presented to the stakeholder group (to include policymakers, practitioners and patients) and their opinions will be sought to direct the dissemination strategy for these groups, with the aim of ensuring that important recommendations reach the appropriate decision-makers. We will endeavour in particular to reach policy makers and researchers engaged in the development and evaluation of interventions designed to reduce variation in test ordering, in order that future work in this area can be informed by the new knowledge generated in this review. We envision the production of user-friendly and accessible summaries of the findings and our recommendations and the use of existing networks and social media to promote these outputs to help ensure maximum visibility.

Author contributions

CD conceived the study with input from GW. CD wrote the first draft of this manuscript and GW provided criticism and refinement. CD and GW approved the final version.

Funding statement

Claire Duddy (CD) is funded by a National Institute of Health Research (NIHR) Research Methods Programme Systematic Review Fellowship (NIHR-RM-SR-2017-08-018). Geoff Wong (GW) and Trish Greenhalgh (TG) are supporting CD in this project. TG is partly funded by the National Institute for Health Research Biomedical Research Centre, Oxford, grant BRC-1215-20008 to the Oxford University Hospitals NHS Foundation Trust and the University of Oxford. GW is partly supported by the Evidence Synthesis Working Group of the NIHR School of Primary Care [Project Number 390].

The views expressed in this publication are those of the author(s) and not necessarily those of the NHS, the National Institute for Health Research or the Department of Health.

Competing interests

CD and GW are both members of the Royal College of General Practitioners (United Kingdom) Overdiagnosis and Overtreatment Group.

GW is an NHS General Practitioner and joint Deputy Chair of the NIHR Health Technology Assessment Out-of-Hospital Panel.

REFERENCES

1 Larsson A, Palmer M, Hultén G, *et al.* Large Differences in Laboratory Utilisation between Hospitals in Sweden. *Clin Chem Lab Med* 2000;**38**:383–9.https://www.degruyter.com/view/j/cclm.2000.38.issue-5/cclm.2000.056/cclm.2000.056.xml (accessed 19 Dec 2017).

2 Verstappen W, Ter Riet G, Dubois WI, *et al.* Variation in test ordering behaviour of GPs: professional or context-related factors? *Fam Pract* 2004;**21**:387–95. doi:10.1093/fampra/cmh408

3 Davis P, Gribben B, Lay-Yee R, *et al.* How much variation in clinical activity is there between general practitioners? A multi-level analysis of decision-making in primary care.

For peer review only

J Health Serv Res Policy 2002;**7**:202–8. doi:10.1258/135581902320432723
4 Taroni F, Siass R, Traversa G, *et al.* The nature, content and interpractice variation of general practice: A regional study in Italy. *Eur J Epidemiol* 1990;**6**:313–8. doi:10.1007/BF00150439
5 Hartley RM, Charlton JR, Harris CM, *et al.* Patterns of physicians' use of medical resources in ambulatory settings. *Am J Public Health* 1987;**77**:565–7. doi:10.2105/AJPH.77.5.565
6 Verstappen W, Ter Riet G, Van Der Weijden T, *et al.* Variation in requests for imaging investigations by general practitioners: A multilevel analysis. *J Health Serv Res Policy* 2005;**10**:25–30. doi:10.1177/135581960501000107
7 Powell EC, Hampers LC. Physician Variation in Test Ordering in the Management of Gastroenteritis in Children. *Arch Pediatr Adolesc Med* 2003;**157**:978. doi:10.1001/archpedi.157.10.978
8 Chami N, Simons JE, Sweetman A, *et al.* Rates of inappropriate laboratory test utilization in Ontario. *Clin Biochem* 2017;**50**:822–7. doi:10.1016/J.CLINBIOCHEM.2017.05.004
9 Lin DC, Straseski JA, Schmidt RL. Multicenter Benchmark Study Reveals Significant Variation in Thyroid Testing in the United States. *Thyroid* 2017;**27**:1232–45. doi:10.1089/thy.2017.0190
10 *The 2nd Atlas of Variation in NHS Diagnostic Services in England Reducing unwarranted variation to improve health outcomes and value*. 2017. www.gov.uk/phe
11 Sá L, Teixeira ASC, Tavares F, *et al.* Diagnostic and laboratory test ordering in Northern Portuguese Primary Health Care: a cross-sectional study. *BMJ Open* 2017;**7**:e018509. doi:10.1136/bmjopen-2017-018509
12 O'Sullivan JW, Albasri A, Nicholson BD, *et al.* Overtesting and undertesting in primary care: a systematic review and meta-analysis. *BMJ Open* 2018;**8**:e018557. doi:10.1136/bmjopen-2017-018557
13 *The NHS Atlas of Variation in Diagnostic Services*. 2013. https://fingertips.phe.org.uk/profile/atlas-of-variation
14 Zhi M, Ding EL, Theisen-Toupal J, *et al.* The Landscape of Inappropriate Laboratory Testing: A 15-Year Meta-Analysis. *PLoS One* 2013;**8**:e78962. doi:10.1371/journal.pone.0078962
15 Busby J, Schroeder K, Woltersdorf W, *et al.* Temporal growth and geographic variation in the use of laboratory tests by NHS general practices: using routine data to identify research priorities. *Br J Gen Pr* 2013;**63**:e256-66. doi:10.3399/bjgp13X665224
16 van Walraven C, Naylor CD. Do We Know What Inappropriate Laboratory Utilization Is? A Systematic Review of Laboratory Clinical Audits. *JAMA* 1998;**12**:550–8.https://jamanetwork.com/journals/jama/fullarticle/vol/280/pg/550 (accessed 20 Dec 2017).
17 Hauser RG, Shirts BH. Do We Now Know What Inappropriate Laboratory Utilization Is? An Expanded Systematic Review of Laboratory Clinical Audits. *Am J Clin Pathol* 2014;**141**:774–83. doi:10.1309/AJCPX1HIEM4KLGNU
18 Lord Carter of Coles. Report of the review of NHS pathology services in England. 2006. http://webarchive.nationalarchives.gov.uk/20130123210254/http://www.dh.gov.uk/en/Publicationsandstatistics/Publications/PublicationsPolicyAndGuidance/DH_4137606 (accessed 15 Feb 2018).
19 Hofmann B. Diagnosing overdiagnosis: conceptual challenges and suggested solutions. *Eur J Epidemiol* 2014;**29**:599–604. doi:10.1007/s10654-014-9920-5
20 Raffle AE, Gray JAM. *Screening: evidence and practice*. Oxford University Press 2007.
21 Newman-Toker DE. A unified conceptual model for diagnostic errors: underdiagnosis, overdiagnosis, and misdiagnosis. *Diagnosis* 2014;**1**:43–8. doi:10.1515/dx-2013-0027
22 Carrieri V, Wuebker A. Assessing inequalities in preventative care use in Europe: A

For peer review only

special case of health-care inequalities? 2012. https://www.york.ac.uk/media/economics/documents/herc/wp/12_25.pdf (accessed 9 Mar 2018).
23 Catalogue of Bias Collaboration, Banerjee A, O'Sullivan J, *et al.* Diagnostic access bias. Cat. Biases. 2017.https://catalogofbias.org/biases/diagnostic-access-bias/ (accessed 9 Mar 2018).
24 Neal RD, Allgar VL. Sociodemographic factors and delays in the diagnosis of six cancers: analysis of data from the 'National Survey of NHS Patients: Cancer'. *Br J Cancer* 2005;**92**:1971–5. doi:10.1038/sj.bjc.6602623
25 Moynihan R, Doust J, Henry D. Preventing overdiagnosis: How to stop harming the healthy. *BMJ* 2012;**344**:e3502. doi:10.1136/bmj.e3502
26 Woolf SH, Harris R. The harms of screening: New attention to an old concern. *JAMA* 2012;**307**:565–6.https://doi.org/10.1001/jama.2012.100 (accessed 6 Dec 2017).
27 Copp T, Jansen J, Doust J, *et al.* Are expanding disease definitions unnecessarily labelling women with polycystic ovary syndrome? *BMJ* 2017;**358**:j3694. doi:10.1136/bmj.j3694
28 Moynihan R, Glassock R, Doust J. Chronic kidney disease controversy: How expanding definitions are unnecessarily labelling many people as diseased. *BMJ* 2013;**347**:f4298. doi:10.1136/bmj.f4298
29 Berwick DM, Hackbarth AD. Eliminating waste in US health care. *JAMA* 2012;**307**:1513–6. doi:10.1001/jama.2012.362
30 Newman-Toker DE, McDonald KM, Meltzer DO. How much diagnostic safety can we afford, and how should we decide? A health economics perspective. *BMJ Qual Saf* 2013;**22**:ii11-ii20. doi:10.1136/bmjqs-2012-001616
31 Glasziou P, Moynihan R, Richards T. Too much medicine ; too little care. *BMJ* 2013;**347**:1–2. doi:10.1136/bmj.f4247
32 BMJ. Too Much Medicine. http://www.bmj.com/too-much-medicine
33 Choosing Wisely UK. http://www.choosingwisely.co.uk
34 Moynihan R, Cassels A. *Selling Sickness: How Drug Companies Are Turning Us All Into Patients*. Crows Nest, NSW: : Allen & Unwin 2005.
35 Welch H, Schwartz L, Woloshin S. *Overdiagnosed: Making People Sick in the Pursuit of Health*. Boston: : Beacon Press 2011.
36 McCartney M. *The Patient Paradox: Why Sexed-Up Medicine is Bad For Your Health*. Great Britain: : Pinter & Martin 2013.
37 Welch H. *Less Medicine, More Health: 7 Assumptions that Drive Too Much Medical Care*. Boston: : Beacon Press 2015.
38 Edwards M. Doctors 'over-diagnose'. Telegraph. 2010.http://www.telegraph.co.uk/news/health/8008608/Doctors-over-diagnose.html
39 Welch H. If you feel O.K., Maybe You Are O.K. New York Times. 2012.http://www.nytimes.com/2012/02/28/opinion/over-diagnosis-as-a-flaw-in-health-care.html
40 Gawande A. Overkill. New Yorker. 2015.http://www.newyorker.com/magazine/2015/05/11/overkill-atul-gawande
41 Srivastava R. Do I really need 'the test'? Too many tests could do patients more harm than good. Guardian. 2016.http://www.theguardian.com/commentisfree/2016/sep/07/do-i-really-need-the-test-too-many-tests-could-do-patients-more-harm-than-good
42 Pathirana T, Clark J, Moynihan R. Mapping the drivers of overdiagnosis to potential solutions Searching the literature. *BMJ* 2017;**358**. doi:10.1136/bmj.j3879
43 Carter SM, Rogers W, Heath I, *et al.* The challenge of overdiagnosis begins with its definition. *BMJ* 2015;**350**:h869. doi:10.1136/bmj.h869
44 Rogers WA, Mintzker Y. Getting clearer on overdiagnosis. *J Eval Clin Pract* 2016;**22**:580–

For peer review only

7. doi:10.1111/jep.12556
45 Welch HG, Black WC. Overdiagnosis in cancer. *J Natl Cancer Inst* 2010;**102**:605–13. doi:10.1093/jnci/djq099
46 Moynihan R, Heath I, Henry D. Selling sickness: the pharmaceutical industry and disease mongering. *BMJ* 2002;**324**:886–91. doi:10.1136/bmj.324.7342.886
47 McCaffery KJ, Jansen J, Scherer LD, *et al.* Walking the tightrope: Communicating overdiagnosis in modern healthcare. *BMJ* 2016;**352**:i348. doi:10.1136/bmj.i348
48 Hofmann B. Defining and evaluating overdiagnosis. *J Med Ethics* 2016;**42**:715–6. doi:10.1136/medethics-2016-103716
49 Deyo RA. Cascade Effects of Medical Technology. *Annu Rev Public Health* 2002;**23**:23–44. doi:10.1146/annurev.publhealth.23.092101.134534
50 Carpenter CR, Raja AS, Brown MD. Overtesting and the downstream consequences of overtreatment: Implications of 'preventing overdiagnosis' for emergency medicine. *Acad Emerg Med* 2015;**22**:1484–92. doi:10.1111/acem.12820
51 Hofmann B, Welch HG. New diagnostic tests: more harm than good. *BMJ* 2017;**358**:j3314. doi:10.1136/bmj.j3314
52 Fisher ES, Welch HG. Avoiding the Unintended Consequences of Growth in Medical Care. *JAMA* 1999;**281**:446–53. doi:10.1001/jama.281.5.446
53 Harris RP, Sheridan SL, Lewis CL, *et al.* The harms of screening : A proposed taxonomy and application to lung cancer screening. *JAMA Intern Med* 2014;**174**:281–6. doi:10.1001/jamainternmed.2013.12745
54 Czoski-Murray C, Lloyd Jones M, McCabe C, *et al.* What is the value of routinely testing full blood count, electrolytes and urea, and pulmonary function tests before elective surgery in patients with no apparent clinical indication and in subgroups of patients with common comorbidities: a systematic review of the clinical and cost-effective literature. *Health Technol Assess (Rockv)* 2012;**16**. doi:10.3310/hta16500
55 Beilby J, Silagy C. Trials of providing costing information to general practitioners: a systematic review. *Med J Aust* 1997;**167**:89–92.
56 Georgiou A, Williamson M, Westbrook JI, *et al.* The impact of computerised physician order entry systems on pathology services: A systematic review. *Int J Med Inform* 2007;**76**:514–29. doi:10.1016/J.IJMEDINF.2006.02.004
57 Ho J, Chau J, Cheung N. Effectiveness of emergency nurses' use of the Ottowa Ankle Rules to initiate radiographic tests on improving healthcare outcomes for patients with ankle injuries: a systematic review. *Int J Nurs Stud* 2016;**63**:37–47. doi:10.1016/j.ihnurstu.2016.08.016
58 Thomas RE, Vaska M, Naugler C, *et al.* Interventions at the laboratory level to reduce laboratory test ordering by family physicians: Systematic review. *Clin Biochem* 2015;**48**:1358–65. doi:10.1016/J.CLINBIOCHEM.2015.09.014
59 Ivers N, Jamtvedt G, Flottorp S, *et al.* Audit and feedback: effects on professional practice and healthcare outcomes. *Cochrane Database Syst Rev* 2012;:CD000259. doi:10.1002/14651858.CD000259.pub3
60 Main C, Moxham T, Wyatt JC, *et al.* Computerised decision support systems in order communication for diagnostic, screening or monitoring test ordering: Systematic reviews of the effects and cost-effectiveness of systems. *Health Technol Assess (Rockv)* 2010;**14**:1–227. doi:10.3310/hta14480
61 Georgiou A, Prgomet M, Markewycz A, *et al.* The impact of computerized provider order entry systems on medical-imaging services: a systematic review. *J Am Med Informatics Assoc* 2011;**18**:335–40. doi:10.1136/amiajnl-2010-000043
62 Roshanov PS, You JJ, Dhaliwal J, *et al.* Can computerized clinical decision support systems improve practitioners' diagnostic test ordering behavior? A decision-maker-researcher partnership systematic review for the CCDSS Systematic Review Team.

For peer review only

Implement Sci 2011;**6**. doi:10.1186/1748-5908-6-88
63 Cadogan SL, Browne JP, Bradley CP, *et al.* The effectiveness of interventions to improve laboratory requesting patterns among primary care physicians: a systematic review. *Implement Sci* 2015;**10**. doi:10.1186/s13012-015-0356-4
64 Goldzweig CL, Orshansky G, Paige NM, *et al.* Electronic Health Record–Based Interventions for Improving Appropriate Diagnostic Imaging. *Ann Intern Med* 2015;**162**:557–65. doi:10.7326/M14-2600
65 Kobewka DM, Ronksley PE, Mckay JA, *et al.* Influence of educational, audit and feedback, system based, and incentive and penalty interventions to reduce laboratory test utilization: a systematic review. *Clin Chem Lab Med* 2015;**53**:157–83. doi:10.1515/cclm-2014-0778
66 Thomas RE, Vaska M, Naugler C, *et al.* Interventions to Educate Family Physicians to Change Test Ordering. *Acad Pathol* 2016;**3**:237428951663347. doi:10.1177/2374289516633476
67 Delvaux N, Thienen K Van, Heselmans A, *et al.* The Effects of Computerized Clinical Decision Support Systems on Laboratory Test Ordering A Systematic Review. *Arch Pathol Lab Med* 2017;**141**:585–95.http://www.archivesofpathology.org/doi/pdf/10.5858/arpa.2016-0115-RA (accessed 20 Dec 2017).
68 Sood R, Sood A, Ghosh AK. Non-evidence-based variables affecting physicians' test-ordering tendencies: a systematic review. *Neth J Med* 2007;**65**:167–77.http://www.njmonline.nl/getpdf.php?id=529 (accessed 20 Dec 2017).
69 Eisenberg JM. Physician Utilization: The State of Research about Physicians' Practice Patterns. *Med Care* 1985;**23**:461–83. doi:10.2307/3764984
70 Leurquin P, Van Casteren V, De Maeseneer J. Use of blood tests in general practice: a collaborative study in eight European countries. Eurosentinel Study Group. *Br J Gen Pract* 1995;**45**:21–5.http://www.ncbi.nlm.nih.gov/pubmed/7779470 (accessed 4 Jan 2018).
71 Wong G, Greenhalgh T, Pawson R. What is a realist review and what can it do for me? An introduction to realist synthesis. 2009.http://pram.mcgill.ca/Wong_G_JUNE09_what_is_a_realist_review_presentation.pdf
72 The RAMESES II Project. Realist evaluation, realist synthesis, realist research - what's in a name? 2017. http://www.ramesesproject.org/media/RAMESES_II_RE_RS_RR_whats_in_a_name.pdf (accessed 31 May 2018).
73 Pawson R. Realist Methodology: The Building Blocks of Evidence. In: *Evidence-based policy: a realist perspective*. London: : SAGE Publications Ltd 2006. 17–37. doi:10.4135/9781849209120
74 Pawson R. Invisible Mechanisms I; The Long Road to Behavioural Change. In: *The Science of Evaluation: A Realist Manifesto*. London: : SAGE Publications Ltd 2013. 115–37. doi:10.4135/9781473913820
75 Pawson R, Greenhalgh T, Harvey G, *et al.* Realist review - a new method of systematic review designed for complex policy interventions. *J Health Serv Res Policy* 2005;**10**:21–34. doi:10.1258/1355819054308530
76 Pawson R. First Principles: A Realist Diagnostic Workshop. In: *The Science of Evaluation: A Realist Manifesto*. London: : SAGE Publications Ltd 2013. 13–28.
77 Marchal B, Van Belle S, Westhorp G, *et al.* Realist Evaluation. Better Eval. 2015.https://www.betterevaluation.org/en/approach/realist_evaluation (accessed 31 May 2018).
78 Wong G. 'Wrong diagnosis, wrong treatment': why researchers and clinicians should question their assumptions. NIHR Blog. 2017.https://www.nihr.ac.uk/blogs/wrong-

For peer review only

diagnosis-wrong-treatment-why-researchers-and-clinicians-should-question-their-assumptions/7374
79 Papoutsi C, Mattick K, Pearson M, *et al.* Social and professional influences on antimicrobial prescribing for doctors-in-training: a realist review. *J Antimicrob Chemother* 2017;**72**:2418–30. doi:10.1093/jac/dkx194
80 Hofmann BM. Too much technology. *BMJ* 2015;**350**:h705. doi:10.1136/BMJ.H705
81 Petticrew M, Rehfuess E, Noyes J, *et al.* Synthesizing evidence on complex interventions: how meta-analytical, qualitative, and mixed-method approaches can contribute. *J Clin Epidemiol* 2013;**66**:1230–43. doi:10.1016/j.jclinepi.2013.06.005
82 Pawson R. A Complexity Checklist. In: *The Science of Evaluation: A Realist Manifesto*. London: : SAGE Publications Ltd 2013. 33–46. doi:10.4135/9781473913820
83 Pawson R. Informed Guesswork: The Realist Response to Complexity. In: *The Science of Evaluation: A Realist Manifesto*. London: : SAGE Publications Ltd 2013. 81–112. doi:10.4135/9781473913820
84 Pawson R, Greenhalgh T, Harvey G, *et al.* Realist synthesis: an introduction. 2004. https://www.researchgate.net/profile/Gill_Harvey/publication/228855827_Realist_Synthesis_An_Introduction/links/0fcfd507f0b7cbb2ce000000/Realist-Synthesis-An-Introduction.pdf (accessed 7 Dec 2017).
85 Pawson R. Realist Synthesis: New Protocols for Systematic Review. In: *Evidence-based policy: a realist perspective*. London: : SAGE Publications Ltd 2006. 73–104. doi:10.4135/9781849209120
86 RAMESES Project Team. *RAMESES Quality Standards For Realist Synthesis (for researchers and peer-reviewers)*. 2014. http://www.ramesesproject.org/media/RS_qual_standards_researchers.pdf (accessed 7 Dec 2017).
87 Wong G, Greenhalgh T, Westhorp G, *et al.* RAMESES publication standards: realist syntheses. *BMC Med* 2013;**11**:21. doi:10.1186/1741-7015-11-21
88 Booth A. Systematic searching for theory to inform systematic reviews: is it feasible? Is it desirable? *Health Info Libr J* 2015;**32**:220–35.http://ovidsp.ovid.com/ovidweb.cgi?T=JS&PAGE=reference&D=emed17&NEWS=N&AN=607682447 (accessed 7 Dec 2017).
89 Booth A, Harris J, Croot E, *et al.* Towards a methodology for cluster searching to provide conceptual and contextual 'richness'; for systematic reviews of complex interventions: case study (CLUSTER). *BMC Med Res Methodol* 2013;**13**. doi:10.1186/1471-2288-13-118
90 Greenhalgh T, Peacock R. Effectiveness and Efficiency of Search Methods in Systematic Reviews of Complex Evidence: Audit of Primary Sources. *BMJ* 2005;**331**:1064–5. doi:10.1136/bmj.38636.593461.68
91 Pawson R, Tilley N. How to Design a Realistic Evaluation. In: *Realistic Evaluation*. London: : SAGE Publications Ltd 1997. 83–114.
92 Wong G, Westhorp G, Pawson R, *et al. Realist Synthesis: RAMESES Training Materials*. 2013. http://www.ramesesproject.org/media/Realist_reviews_training_materials.pdf (accessed 7 Dec 2017).
93 Alger I, Salanié F. A theory of fraud and overtreatment in experts markets. *J Econ Manag Strateg* 2006;**15**:853–81. doi:10.1111/j.1530-9134.2006.00120.x
94 Hozo I, Djulbegovic B. When Is Diagnostic Testing Inappropriate or Irrational? Acceptable Regret Approach. *Med Decis Mak* 2008;**28**:540–53. doi:10.1177/0272989X08315249
95 Djulbegovic B, van den Ende J, Hamm RM, *et al.* When is rational to order a diagnostic test, or prescribe treatment: The threshold model as an explanation of practice variation. *Eur J Clin Invest* 2015;**45**:485–93. doi:10.1111/eci.12421
96 Hozo I, Tsalatsanis A, Djulbegovic B. Expected utility versus expected regret theory

For peer review only

versions of decision curve analysis do generate different results when treatment effects are taken into account. *J Eval Clin Pract* Published Online First: 2016. doi:10.1111/jep.12676
97 Woodward RS, Schnitzler MA, Kvols LK. Reduced uncertainty as a diagnostic benefit: An initial assessment of somatostatic receptor scintigraphy's value in detecting distant metastases of carcinoid liver tumours. *Health Econ* 1998;**7**:149–60.
98 Croskerry P, Petrie DA, Reilly JB, *et al.* Deciding about fast and slow decisions. *Acad Med* 2014;**89**:197–200. doi:10.1097/ACM.0000000000000121
99 Aspinall P, Hill AR. Clinical Inferences and Decisions-II. Decision Trees, Receiver Operator Curves and Subjective Probability. *Ophthalmic Physiol Opt* 1984;**4**:31–8. doi:10.1111/j.1475-1313.1984.tb00329.x
100 Nilsen P, Roback K, Broström A, *et al.* Creatures of habit: accounting for the role of habit in implementation research on clinical behaviour change. *Implement Sci* 2012;**7**.https://implementationscience.biomedcentral.com/articles/10.1186/1748-5908-7-53 (accessed 21 Dec 2017).
101 Zaat JOM, van Eijk JTM, Bonte HA. Laboratory Test Form Design Influences Test Ordering by General Practitioners in the Netherlands. *Med Care* 1992;**30**:189–98.http://www.jstor.org/stable/3766214 (accessed 21 Dec 2017).
102 Van Bokhoven MA, Koch H, Van Der Weijden T, *et al.* The effect of watchful waiting compared to immediate test ordering instructions on general practitioners' blood test ordering behaviour for patients with unexplained complaints; a randomized clinical trial (ISRCTN55755886). *Implement Sci* 2012;**7**. doi:10.1186/1748-5908-7-29
103 Verstappen WHJM, Van Der Weijden T, Dubois WI, *et al.* Improving test ordering in primary care: The added value of a small-group quality improvement strategy compared with classic feedback only. *Ann Fam Med* 2004;**2**:569–75. doi:10.1370/afm.244
104 Morgan S, Saltis T, Coleman J, *et al.* Test result audit and feedback (TRAFk) as a supervision method for rational test ordering in general practice training. *Aust Fam Physician* 2016;**45**:518–22.https://www.racgp.org.au/download/Documents/AFP/2016/July/AFP-July-Research-Morgan.pdf (accessed 21 Dec 2017).
105 Trietsch J, van Steenkiste B, Grol R, *et al.* Effect of audit and feedback with peer review on general practitioners' prescribing and test ordering performance: a cluster-randomized controlled trial. *BMC Fam Pract* 2017;**18**. doi:10.1186/s12875-017-0605-5
106 Poley MJ, Edelenbos KI, Mosseveld M, *et al.* Cost Consequences of Implementing an Electronic Decision Support System for Ordering Laboratory Tests in Primary Care: Evidence from a Controlled Prospective Study in The Netherlands. *Clin Chem* 2007;**53**:213–9. doi:10.1373/clinchem.2006.073908
107 Martins CMS, da Costa Teixeira AS, de Azevedo LFR, *et al.* The effect of a test ordering software intervention on the prescription of unnecessary laboratory tests - a randomized controlled trial. *BMC Med Inform Decis Mak* 2017;**17**:20. doi:10.1186/s12911-017-0416-6
108 Wong G, Greenhalgh T, Westhorp G, *et al.* Realist methods in medical education research: What are they and what can they contribute? *Med Educ* 2012;**46**:89–96. doi:10.1111/j.1365-2923.2011.04045.x
109 Moher D, Liberati A, Tetzlaff J, *et al.* Preferred Reporting Items for Systematic Reviews and Meta-Analyses: The PRISMA Statement. *PLoS Med* 2009;**6**:e1000097. doi:10.1371/journal.pmed.1000097
110 Pawson R. Digging for Nuggets: How 'Bad' Research Can Yield 'Good' Evidence. *Int J Soc Res Methodlogy* 2006;**9**:1364–5579. doi:10.1080/13645570600595314
111 Wong G, Brennan N, Mattick K, *et al.* Interventions to improve antimicrobial prescribing of doctors in training: the IMPACT (IMProving Antimicrobial presCribing of doctors in Training) realist review. *BMJ Open* 2015;**5**:e009059. doi:10.1136/bmjopen-2015-009059

For peer review only

112 RAMESES II Project. Retroduction in realist evaluation. 2017.http://www.ramesesproject.org/media/RAMESES_II_Retroduction.pdf (accessed 22 Nov 2017).
113 Preventing Overdiagnosis. http://www.preventingoverdiagnosis.net
114 International Realist Conference. http://realist2017.org

Figure legends

Figure 1: Steps taken in test ordering decisions
Figure 2: Review project design
Figure 3: Contexts, reasons for test-ordering and range of outcomes
Figure 4: Example CMOCs showing the possible effect of introducing reviews of test ordering behaviour

For peer review only

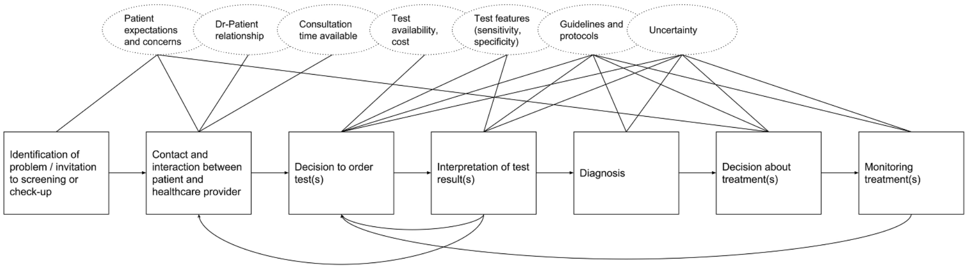

Figure 1: Steps taken in test ordering decisions

276x90mm (300 x 300 DPI)

For peer review only

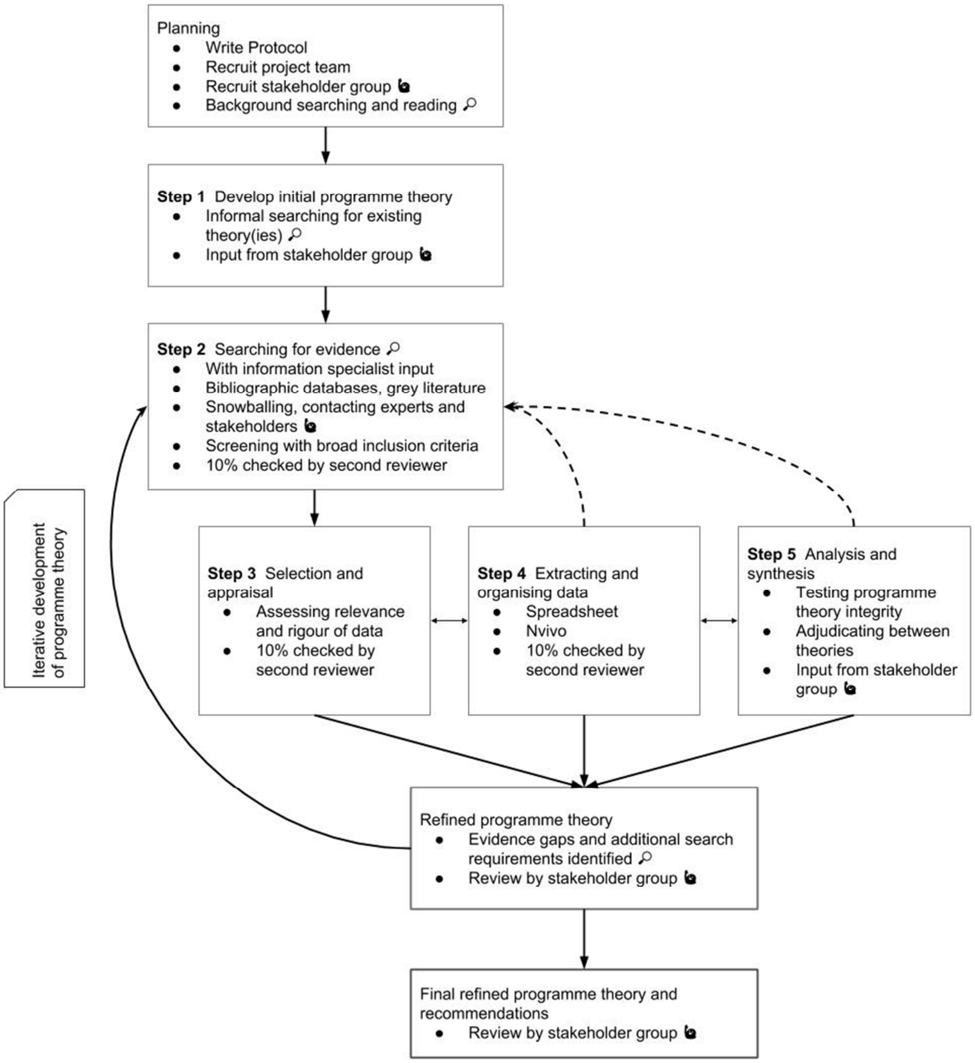

Figure 2: Review project design

83x90mm (300 x 300 DPI)

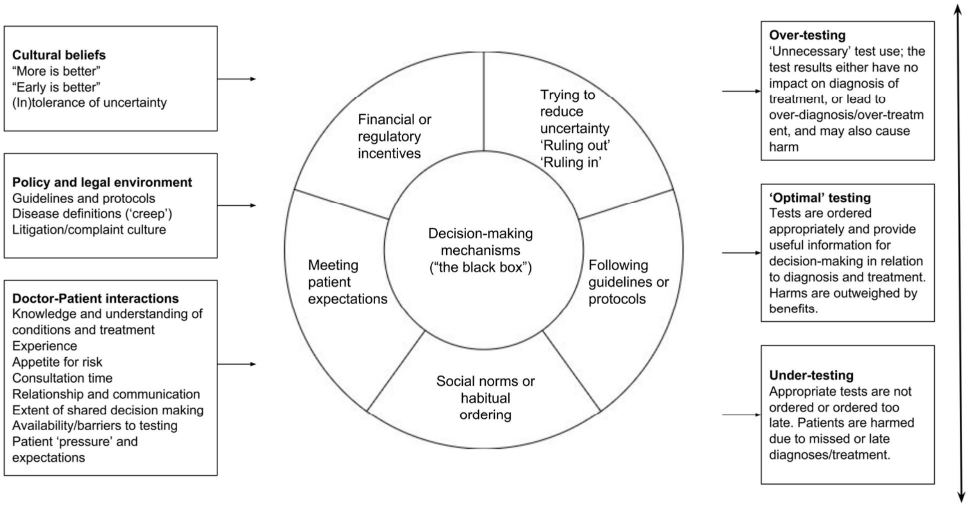

Figure 3: Contexts, reasons for test-ordering and range of outcomes

90x47mm (600 x 600 DPI)

For peer review only

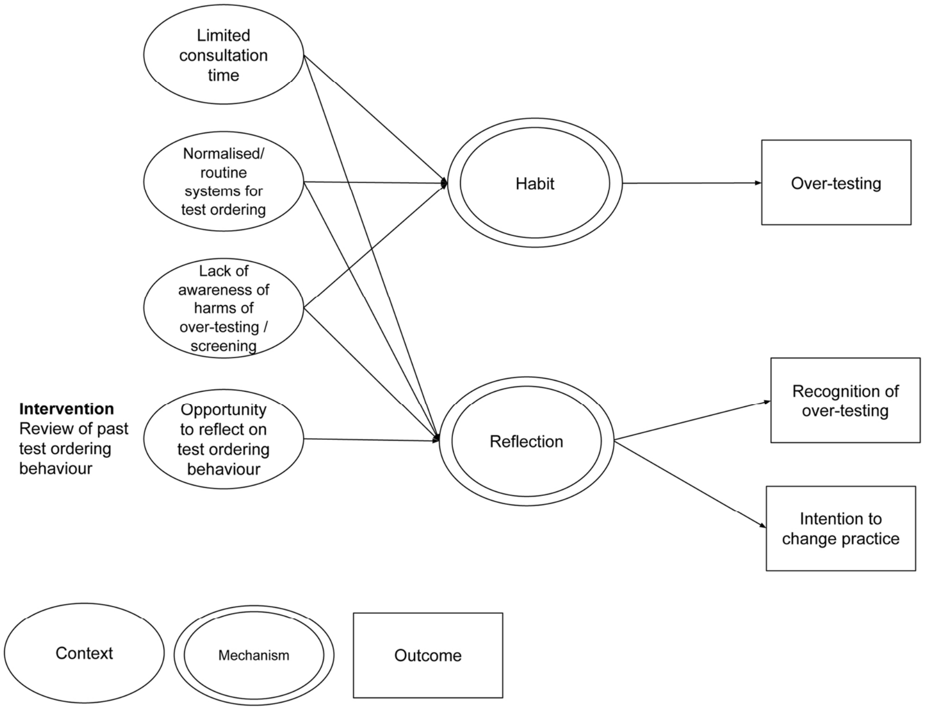

Figure 4: Example CMOCs showing the possible effect of introducing reviews of test ordering behaviour

90x67mm (600 x 600 DPI)

Growth in ‘overdiagnosis’ literature

This chart shows the dramatic increase in published literature on the topic of ‘overdiagnosis’ since the late 2000s as awareness of the problem has grown.

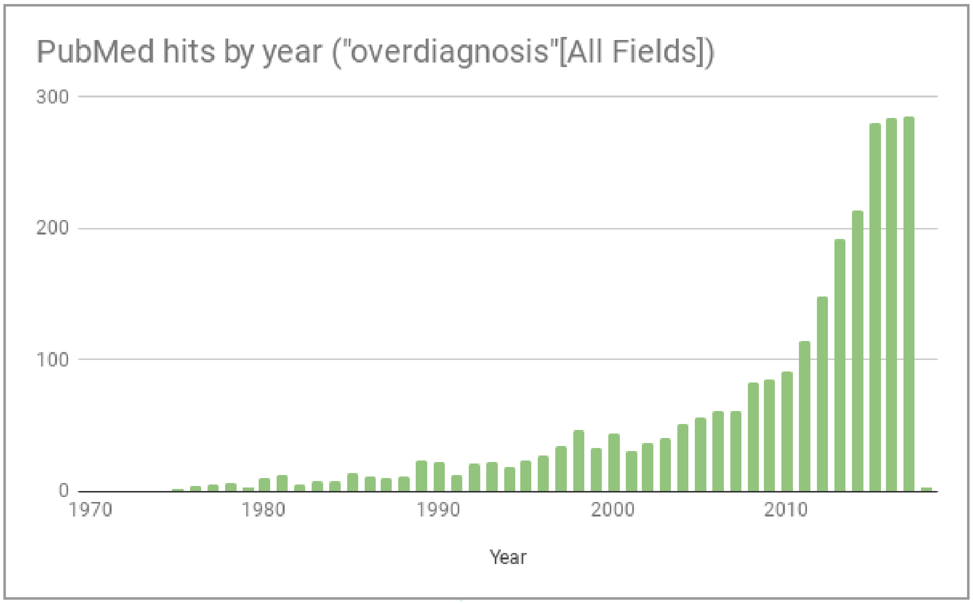

A specific MeSH term, “Medical Overuse”, covering overdiagnosis, overtreatment and related ‘too much medicine’ concepts was introduced in 2016, another sign of increasing interest (previously articles on this topic were indexed as part of the broader “Unnecessary Procedures” category (introduced in 1997).

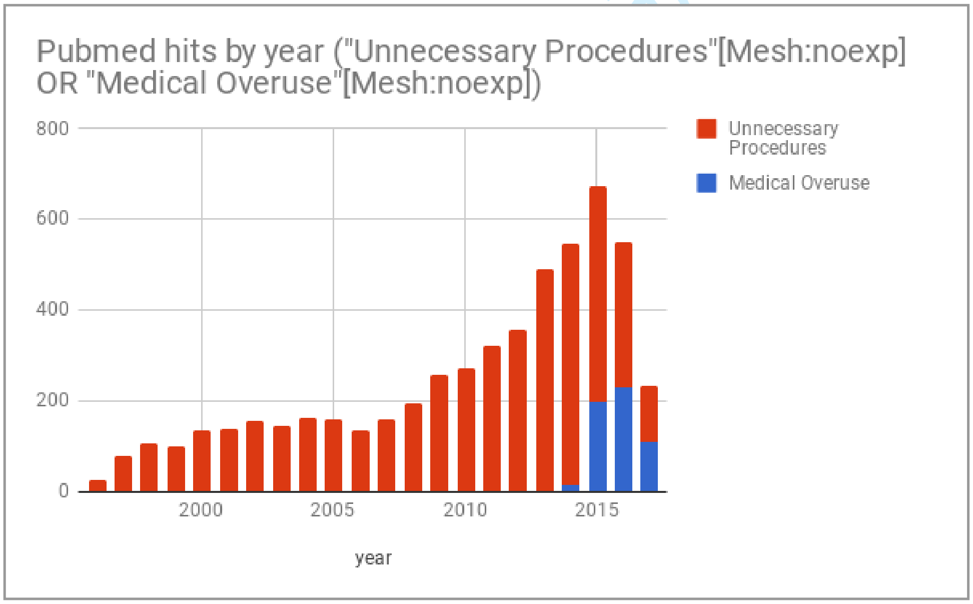

Glossary of realist terminology

(adapted from Realist Synthesis: RAMESES Training Materials)[1]

CMOC	"Context-mechanism-outcome configuration"; a diagrammatic or narrative description offering an explanation of the relationship between some particular context(s), mechanism(s) and outcome(s).<br><br>Multiple CMOCs may exist within a single programme theory.
Context	"Any condition that triggers and/or modifies the behaviour of a mechanism"[2]<br><br>Context refers to the important feature(s) of the circumstances in which a programme exists (or a phenomenon happens) which 'trigger' the mechanisms that generate outcomes. Changes in context over time or in different settings will affect whether and which mechanisms are in operation.
Demi-regularity	A semi-predictable pattern, such as we expect to observe in outcomes that depend on human choice or agency.[2]
Folk theory	An informal theory describing how an intervention is assumed or known to work, or how a phenomenon is assumed or known to come about, according to those practitioners or participants directly involved
Mechanism (generative)	The underlying process by which outcomes are generated. Mechanisms are usually descriptions of the tendencies, reasoning and behaviour of agents involved in a process or participants in a programme and their response to the important context(s) in which they exist.<br><br>Mechanisms are distinguished from "variables or correlates"[3] that are associated with particular outcomes; instead they offer an explanation for why and how observed outcomes happen.
Middle-range theory	A theory that is specific enough to be tested (e.g. against secondary evidence in a realist review, or against primary evidence in a realist evaluation), but abstract or generalisable enough to be transferable, and have explanatory value in other situations.
Programme theory	A theory that describes what an intervention comprises and how it is expected to work, or the process by which the outcomes of interest are thought to come about (expressed as a narrative description or in a diagram).<br><br>A realist programme theory is expressed in terms of the relationship between relevant context(s), mechanism(s) and outcome(s) (or CMOCs).
Substantive theory	An existing established theory from any discipline that can be used to help understand the intervention or phenomenon under examination.

For peer review only

Strategies for early theory-seeking searches

PubMed (19/12/2017)

1	((overdiagnos* OR over-diagnos* OR overtreat* OR over-treat* OR over-prescri* OR overprescri* OR "Unnecessary Procedures"[Mesh])) AND ((("logic model" OR "theory of change" OR "theory of action" OR "outcomes chain" OR "program* theory" OR "program* logic" OR "logical framework*")))	16 hits
2	(overtest* OR over-test* OR “inappropriate test*” OR “unnecessary test*” OR “irrational test*”) AND ("logic model" OR "theory of change" OR "theory of action" OR "outcomes chain" OR "program* theory" OR "program* logic" OR "logical framework*")	5 hits
3	((overdiagnos* OR over-diagnos* OR overtreat* OR over-treat* OR over-prescri* OR overprescri* OR "Unnecessary Procedures"[Mesh])) AND (("theor*" OR "framework*" OR "concept*" OR "model*"))	999 hits
4	(overtest* OR over-test* OR “inappropriate test*” OR “unnecessary test*” OR “irrational test*”) AND (theor* OR framework* OR concept* OR model*)	154 hits

Google Scholar (19/12/2017)

(overdiagnosis OR Overtreatment OR overtesting OR "unnecessary test" OR "inappropriate test" OR "irrational test") AND (theory OR framework OR concept* OR model*)
42,100 hits (first 15 pages screened)

(overdiagnosis OR overtreatment OR overtesting) AND ("logic model" OR "theory of change" OR "theory of action" OR "outcomes chain" OR "program theory" OR "program logic" OR "logical framework")
164 hits

References

1 Wong G, Westhorp G, Pawson R, *et al. Realist Synthesis: RAMESES Training Materials*. 2013. http://www.ramesesproject.org/media/Realist_reviews_training_materials.pdf (accessed 7 Dec 2017).
2 Jagosh J, Macaulay AC, Pluye P, *et al.* Uncovering the Benefits of Participatory Research: Implications of a Realist Review for Health Research and Practice. *Milbank Q* 2012;**90**:311–46. doi:10.1111/j.1468-0009.2012.00665.x
3 Pawson R, Tilley N. *Realistic evaluation*. London: : Sage 1997.

For peer review only
